# Environmental and dynamic effects explain how nisin captures membrane-bound lipid II

**DOI:** 10.1038/s41598-020-65522-y

**Published:** 2020-06-01

**Authors:** Irina Panina, Nikolay Krylov, Dmitry Nolde, Roman Efremov, Anton Chugunov

**Affiliations:** 1 Shemyakin-Ovchinnikov Institute of Bioorganic Chemistry, Russian Academy of Sciences, 16/10 Miklukho-Maklaya St., Moscow, 117997 Russia; 2 National Research University Higher School of Economics, Moscow, 101000 Russia; 3 Moscow Institute of Physics and Technology (State University), Dolgoprudny, 141701 Moscow, Oblast Russia

**Keywords:** Computational biophysics, Computational models, Computational science

## Abstract

Antibiotics (AB) resistance is a major threat to global health, thus the development of novel AB classes is urgently needed. Lantibiotics (i.e. nisin) are natural compounds that effectively control bacterial populations, yet their clinical potential is very limited. Nisin targets membrane-embedded cell wall precursor — lipid II — *via* capturing its pyrophosphate group (PPi), which is unlikely to evolve, and thus represents a promising pharmaceutical target. Understanding of exact molecular mechanism of initial stages of membrane-bound lipid II recognition by water-soluble nisin is indispensable. Here, using molecular simulations, we demonstrate that the structure of lipid II is determined to a large extent by the surrounding water-lipid milieu. In contrast to the bulk solvent, in the bilayer only two conformational states remain capable of nisin binding. In these states PPi manifests a unique arrangement of hydrogen bond acceptors on the bilayer surface. Such a “pyrophosphate pharmacophore” cannot be formed by phospholipids, which explains high selectivity of nisin/lipid II recognition. Similarly, the “recognition module” of nisin, being rather flexible in water, adopts the only stable conformation in the presence of PPi analogue (which mimics the lipid II molecule). We establish the “energy of the pyrophosphate pharmacophore” approach, which effectively distinguishes nisin conformations that can form a complex with PPi. Finally, we propose a molecular model of nisin recognition module/lipid II complex in the bacterial membrane. These results will be employed for further study of lipid II targeting by antimicrobial (poly)cyclic peptides and for design of novel AB prototypes.

## Introduction

The widespread misuse of antibiotics (AB) results in fast development of resistance by bacteria, which presents a growing threat to global health. Discovery of new classes of antibacterial agents with alternative mode of action along with grasp of these mechanisms present the primary scientific challenges. There is a class of lanthionine-containing antimicrobial peptides (AMPs) referred to as lantibiotics, which possesses medicinal potential. Nisin, the most studied lantibiotic, was known even before A. Fleming’s penicillin discovery^1^. Although nisin has never been used in clinical practice due to its poor pharmacokinetics, it has been widely utilized as a food preservative since 1953 with no evidence of any resistance development^2–4^.

**Figure 1:**
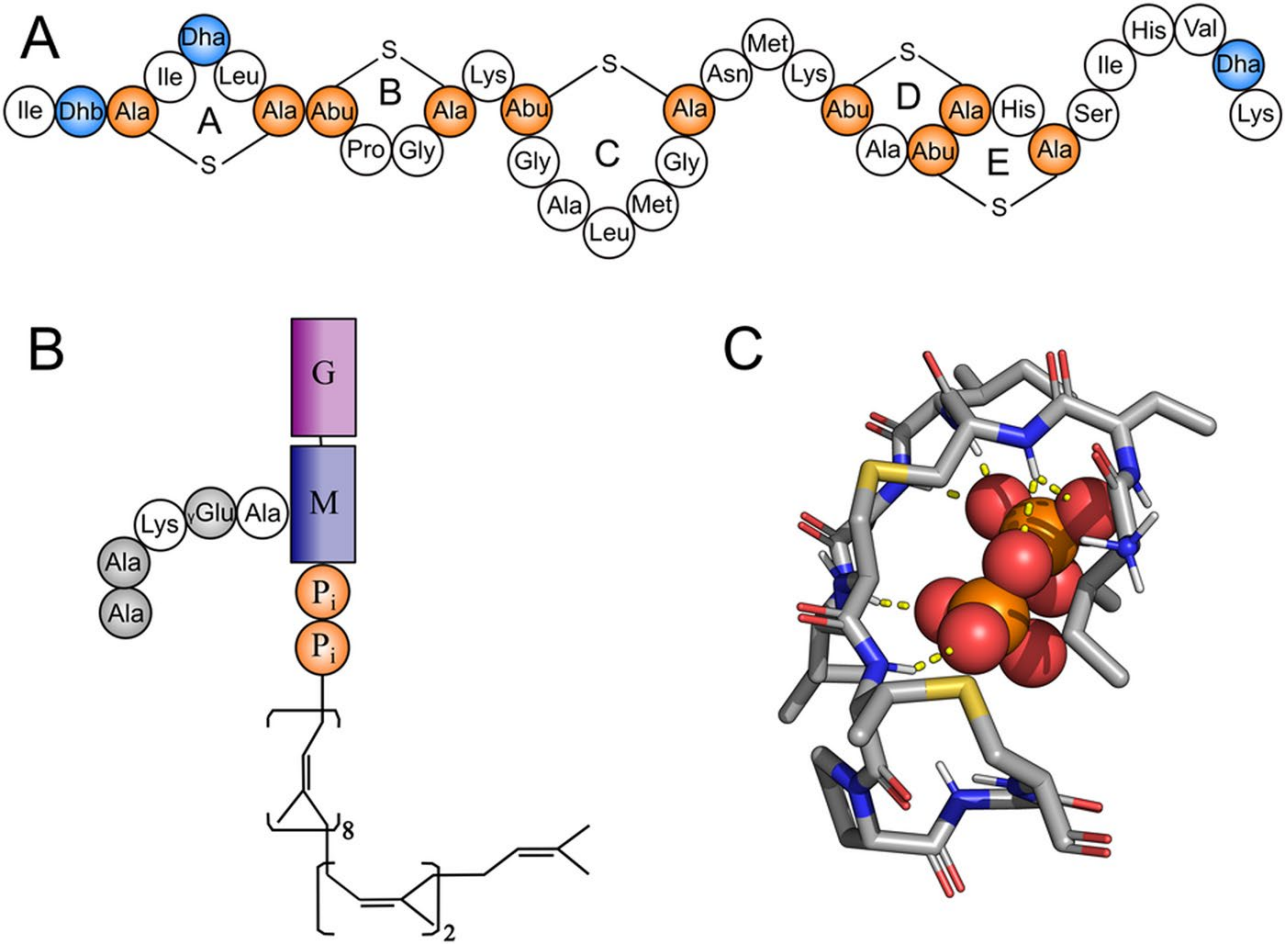
Nisin and lipid II structures, and their complex obtained by NMR in DMSO. **(A)** Schematic structure of nisin. Non-canonical amino acids are colored: unsaturated amino acids dehydroalanine and dehydrobutyrine in *blue*; lantionine and methyl-lantionine in *orange*. **(B)** Schematic structure of lipid II. Sugar moieties N-acetylglucosamine (G) and N-acetylmuramic acid (M) are shown as *magenta* and *purple rectangles*, respectively; D-amino acids in pentapeptide are *grey*; pyrophosphate (PPi) is *orange*.** (C)** NMR structure of nisin/lipid II complex in DMSO solvent^29^.

Nisin is a 34-amino acid peptide produced by certain strains of *Lactococcus Lactis*. Following the ribosomal synthesis, nisin prepeptide undergoes significant post-translational modifications, including proteolytic removal of a leader peptide^5^ and introduction of atypical amino acids, resulting in five lanthionine rings and three unsaturated residues (Fig. 1A)^6^. NMR studies of nisin and its major degradation products under various conditions revealed that the molecule is highly flexible in aqueous solution and consists of two structural domains: an amphiphilic N-terminal fragment including lanthionine rings A, B and C, joint to intertwined D and E lanthionine rings fragment by a “hinge” region^7–9^. Ring A exhibits flexibility in the lanthionine bridge region along with Ile4 and Leu6 side chains^10^. The smallest ring B made up of 4 residues, which also contains Pro9 residue, was shown to have quite limited conformational mobility^11^. Comparative NMR and CD spectra analysis of nisin in water and model micelles indicated that non-aqueous environment does affect the conformation of the N-terminal region^12^. Nisin is primarily active against a broad spectrum of Gram-positive bacteria, however inhibitory activity in combination with a chelating agent against several Gram-negative strains has been reported^13^. Despite being known and used in industry for decades, the intrinsic mechanism of nisin antimicrobial action at atomic level remains rather unclear.

Nisin kills bacteria by cytoplasmic membrane disruption, as well as by inhibiting the cell wall synthesis^5,14^. Possessing overall positive charge +3, nisin binds *in vitro* to lipids with a net negative charge (such as 1,2-dioleoylphosphatidylglycerol (DOPG)) in micromolar concentrations^15^, whereas *in vivo* the minimal inhibitory concentration (MIC) is in the range of nanomoles^16,17^. This increase of efficacy emerges from its interaction with specific membrane-localized target — a bacterial peptidoglycan precursor molecule lipid II; this also facilitates the subsequent pore formation^18,19^.

Since lipid II is the key component of bacterial cell wall biosynthesis^20,21^, it is a target for lantibiotics, glycopeptide antibiotics (such as vancomycin^22,23^), and defensins^24^. Lipid II consists of hydrophilic head group including a peptidoglycan subunit: β-1,4-linked N-acetylglucosamine (GlcNAc) and N-acetylmuramic acid (MurNAc) coupled with a short pentapeptide moiety. The head group is connected to membrane-embedded bactoprenol C55 chain via pyrophosphate (PPi) moiety (Fig. 1B). Being a unique component of prokaryotic cells and essential part of the bacterial cell wall synthesis pathway, lipid II is believed to be a promising target for the next generation of antibiotics — especially given its conserved chemical structure that is unlikely to change due to microbial evolution process.

The N-terminus of nisin molecule (the “recognition module”) interacts with PPi and MurNAc moieties of lipid II, while the C-terminal part (“membrane-active module”) is apparently responsible for pore formation^25^. Binding to lipid II is followed by the assembly of a pore complex with nisin: lipid II stoichiometry of 8:4^19^. Substitutions in the hinge region (residues 20 to 22) result in loss of pore-forming (but not antibacterial) activity^26,27^. Isolated AB rings fragment of nisin retains antimicrobial activity, albeit reduced compared to the full molecule^28^.

Despite the availability of the NMR structure of a complex between nisin and shortened variant of lipid II (Fig. 1C)^29^, the full picture of this recognition is yet to be determined. Although this structure reveals the “pyrophosphate cage” mechanism, in which backbone amides of nisin A and B rings “trap” the PPi moiety, it does not account for the membrane environment, which is native for flexible lipid II molecule. Recent solid-state NMR study^30^ of nisin/lipid II in a membrane-like environment revealed that the “classical” NMR structure of the complex is somewhat misleading, although this work has not delivered a better alternative. At this point, computer simulations provide a functional framework for *in silico* structural studies of lipid II recognition by membrane-active ABs.

At the moment, several molecular dynamics (MD) simulations of lipid II and/or nisin have been performed. Cystine analogues of nisin A and B rings (which are much easier to synthesize) were investigated by MD and displayed good conformational match to the natural peptide^31^. Koch *et al*. performed competitive MD simulations of lipid II in the membrane, either isolated or in complex with nisin, showing that lipid II capture by nisin reduces conformational freedom of the target^32^. Approaching the real stoichiometry^26^, computational study of the ternary nisin_2_/lipid II complex revealed the probable second lipid II binding site at the pentapeptide C-terminal region^33^. The role of non-specific interactions between nisin hinge region and the C-terminus and phospholipids in bacterial membrane deformation was demonstrated by Prince *et al*. in a computational experiment verified by a series of experimental studies^34^.

Our previous MD study of lipid II in phospholipid bilayer^35^ revealed that highly flexible C55 tail of the molecule scans the hydrophobic core of the membrane with overall preferable “L-shape” conformation, where the tail fluctuates between monolayers. We discovered that lipid II (as well as its peptide moiety-deprived analogue) induces long-term perturbations of the model bacterial membrane, which manifests itself as an “amphiphilic pattern” surrounding the lipid II head group at the bilayer surface. We hypothesized that this pattern may guide the target recognition by lantibiotics. Recent Raman spectroscopy data for lipid II in anionic dipalmitoylphosphatidylglycerol (DPPG) or dilauroylphosphatidylglycerol (DLPG) membrane confirm our findings, including local changes of the membrane hydrophobic properties^36^.

The aims of the present study are as follows: 1) to explore the most intimate details of the probable mechanism of lipid II capture by the nisin “recognition module” (rings A and B) by means of MD simulations; 2) to establish the PPi binding pharmacophore. We developed an energy-based scoring function that pinpoints relevant for PPi recognition nisin conformations and may be used for the future design of lipid II capturing drugs. Furthermore, based on the intricacy of conformational ensembles of both partners, we propose the structure of their complex on the bilayer surface, which differs from the well-known NMR complex in DMSO^29^. We emphasize the importance of the correct understanding of the membrane environment and provide the framework for ongoing modeling of lipid II recognition by (poly)cyclic peptides and, as a result, the design of novel antibiotics.

## Results

### Flowchart of the study

Previous *in silico* experiments^35,37^ reveal that PPi group of lipid II is located at the membrane-water interface, and thus is partially inaccessible for extracellular agents such as nisin. This undermines the validity of the molecular model of nisin/lipid II complex, which was obtained by NMR without taking into account the native lipid II environment^29^. Indeed, these doubts are being confirmed by recent NMR study of nisin/lipid II system^30^, which nevertheless did not provide any alternative.

**Figure 2:**
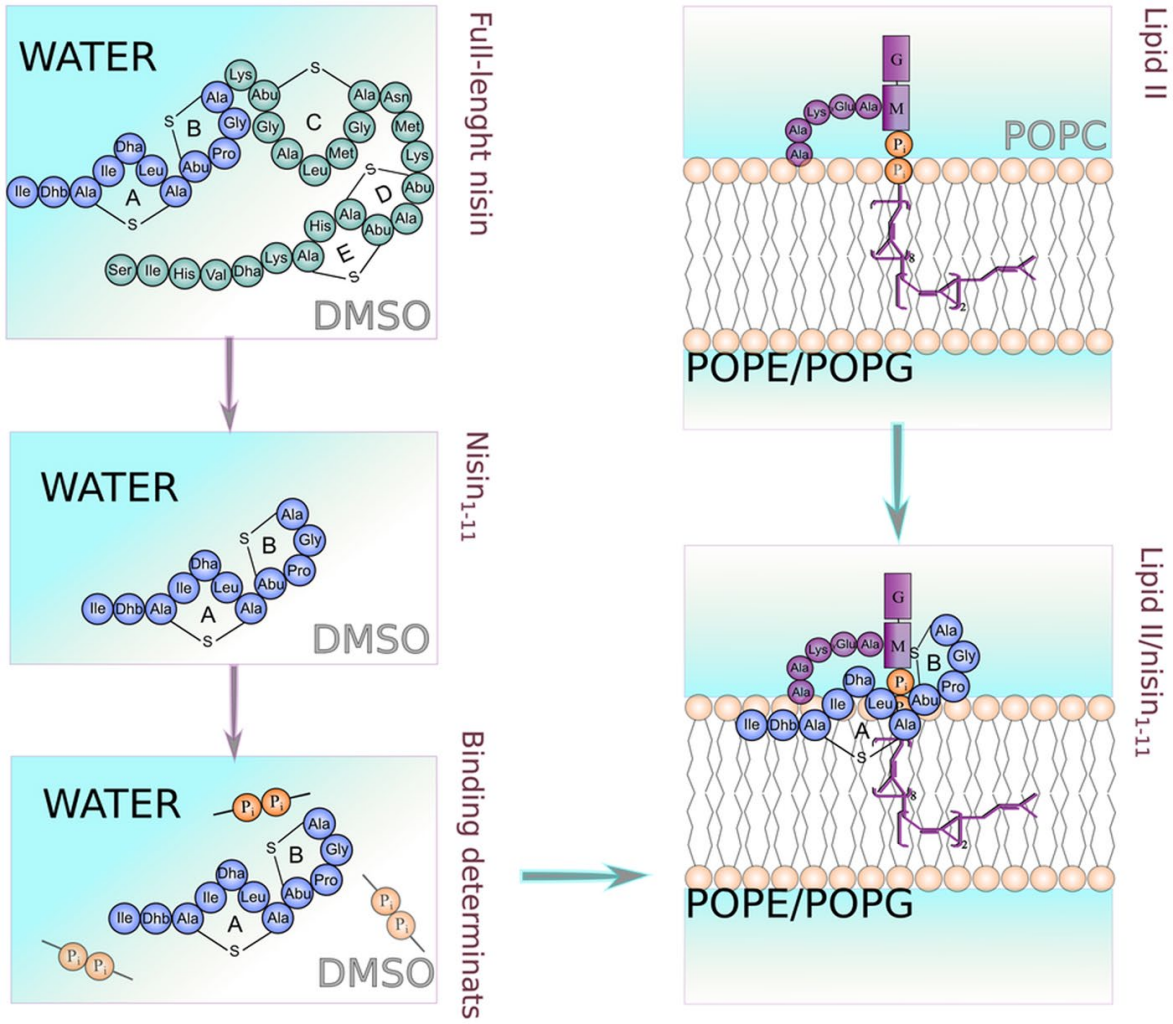
Flowchart of the study. The general coloring scheme is: residues 1–11 of nisin (recognition module; *purple*), residues 12-34 of nisin (membranoactive module; *dark green*), lipid II (*lilac*), PPi (*orange*). For each system the modeling environment is labeled: parent (*black*) and reference (*grey*).

In this paper we aimed to investigate the recognition of lipid II molecule in the most intimate details at several levels of molecular and environmental complexity — from the basic lipid II pharmacophore recognizable by nisin to a realistic model of nisin/lipid II complex in its native bacterial membrane/water environment. The flowchart of the study was as follows (Fig. 2):

1. We searched for the complex-forming conformations of both isolated molecules in their parent environments, i.e. full-length nisin and its N-terminal recognition module (nisin_1–11_) in water solution and lipid II embedded into the model bacterial membrane (mixture of palmitoyl-oleoyl-phosphatidylglycerol with palmitoyl-oleoyl-phosphatidylethanolamine, POPG/POPE). Additional simulations were performed for the molecules in the reference medium: DMSO for nisin and zwitterionic POPC (palmitoyl-oleoyl-phosphatidylcholine) bilayer for lipid II (Fig. 2, upper half).
2. Further, we conducted MD-simulations of nisin_1–11_ (the recognition module) in the presence of PPi analogue in aqueous solution, to study spontaneous complex formation and explore the induced-fit mechanism of this recognition.
3. The discovered pharmacophore prompted us to develop the “energy of the pyrophosphate pharmacophore” (*E*_OO_) computational technique, which helps to effectively identify in MD-trajectory nisin conformations, which can form a complex with PPi.
4. Finally, based on the preferential solvent-induced conformations of the both molecules, we managed to construct a putative complex of nisin recognition module/lipid II in the model bacterial membrane, that remained stable in a long-term MD run.

The full list of the obtained MD trajectories is provided in *Methods*. All these steps are described in detail below.

**Table 1:** *E*_OO_ values for different nisin conformations.

Nisin conformation	*E*_OO_ (kcal/mol)	Additional information
Transient MD complex #1 in water (Fig. 7)	−687.5 ± 38.7	The model has low *E*_OO_ value, however, it is unstable in aqueous environment maybe due to low contact surface area
Non-stable MD complex #2 in water (Fig. 7)	−577.0 ± 35.5	The complex is formed by three N-terminal nisin_1–11_ residues
Stable MD complex #3 in water (Fig. 7)	−682.5 ± 30.1	Nisin_1–11_ in the complex-forming conformation
Stable MD complex #2 in DMSO (Fig. S8)	−761.6 ± 32.2	The similar nisin_1–11_ conformation in water (complex #1, Fig. 7) has higher *E*_OO_ (probably because of rigid NH groups positions in DMSO)
NMR structure in DMSO	−629.1	Model-1 structure from NMR^29^ data
	−643.0 ± 24.8	Averaged value for 20 NMR models

### Overview of nisin and lipid II conformational spaces

#### Overall dynamics of lipid II in the POPG/POPE bilayer

Lipid II behavior in the POPG/POPE bilayer, which was chosen to mimic the membrane of gram-positive bacteria^38^, was described comprehensively in our previous work^35^. The results obtained in the current study entirely reproduce and support all the previous findings, so here we provide only a brief description of this system and focus on the dynamic characteristics of the PPi moiety.

In the course of MD simulations, lipid II head group adopts a wide range of conformations, all “floating” on the bilayer surface. N-acetylglucosamine (G) may be almost fully solvent-exposed (up to 80%) situating right above N-acetylmuramic acid (M) or be buried into membrane leaving only ~20–30% of the surface accessible. Depending on its position, G-sugar may interact *via* hydrogen bonds with either M-sugar, pentapeptide or membrane lipids (~8, 30 and 30% of MD time, respectively). M-sugar forms short-lived H-bonds with PPi group as well as with G-sugar through its -CH_2_-OH group. Except for some minor fluctuations in the distal residues, the peptide displays remarkable stability with Lys3 side chain forming T-like structure with the terminal D-Ala-D-Ala. The latter is highly exposed to solvent, while the remaining pentapeptide residues are partially buried due to their strong binding to phospholipids.

The most flexible lipid II group is the long undecaprenol tail, as indicated by root-mean-square fluctuation (RMSF) values (7 *vs*. 2–5 Å for the rest of the molecule). The same conclusion comes from the analysis of the tail gyration radius (9.3 ± 1.5 Å; Fig. S1). Density profiles indicate that PPi group of lipid II occupies the same bilayer height as phosphate groups (Pi) of phospholipids (Fig. S2).

#### Conformational Dynamics of PPi Group

**Figure 3:**
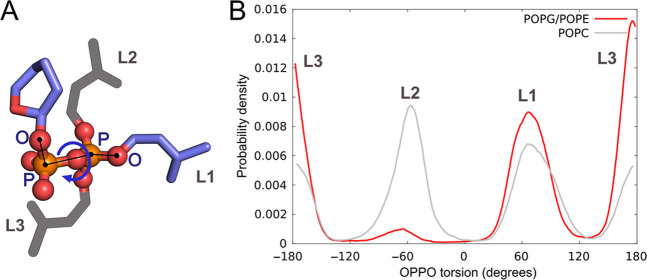
Three major PPi conformations in lipid II obtained via MD simulations in POPG/POPE and POPC bilayers. **(A)** Schematic representation of pseudodihedral angle OPPO and three major conformations of PPi (L1–L3) (see also Fig. S3). **(B)** OPPO distributions in MD trajectories of lipid II in model bacterial membrane (POPG/POPE; *red*; the data are cumulative from three independent MD runs) and in zwitterionic bilayer (POPC; *grey*).

Lipid II’s PPi group in the membrane is located at the lipid–water boundary, as reported previously^35,37^. This study revealed three distinct PPi’s conformations in POPG/POPE membrane, differing by rotation of lipid II head group relative to bacterioprenol tail about P–P axis, which can be quantified by the magnitude of a “pseudodihedral” angle OPPO (Fig. 3A).

Figure 3B shows that the OPPO angle distribution of lipid II in bacterial POPG/POPE membrane has three preferable states: **L1** (OPPO torsion = 68 ± 19°), **L2** ( − 70 ± 19°), and **L3** (176 ± 14°), which correspond to staggered *gauche* (L1 and L2) and *anti*-conformations (L3). Different MD runs exhibit distinct dihedral angle populations. These may result from the limited simulations time and hindered conformational transitions (state lifetime 70–1000 ns, Fig. S4) due to the strong PPi binding to membrane lipids (9–10 H-bonds on average). This interaction is not that strong in the reference POPC (eukaryotic-like) bilayer: just 3–5 H-bonds on average. The inability to form more H-bonds leads to more frequent conformational transitions (15 transitions per 1 μs as compared to just 2–4 in POPG/POPE membrane). The resulting OPPO dihedral distribution profile in POPC bilayer has no pronounced preference for L1, L2 or L3 conformation (Fig. 3B, *grey* plot).

In all simulations PPi moiety remained relatively accessible to the solvent (20–50% of the surface area) during almost the entire MD time. Its moderate conformational fluctuations, re-orientation with respect to the bilayer normal, as well as interaction with polar lipid heads result in “flickering” pattern of PPi H-bond acceptors at the bilayer surface (2–4 solvent-accessible oxygen atoms at time), which certainly affects its visibility for the extracellular agents such as nisin.

#### PPi group in lipid II manifests unique O–O pattern

**Figure 4:**
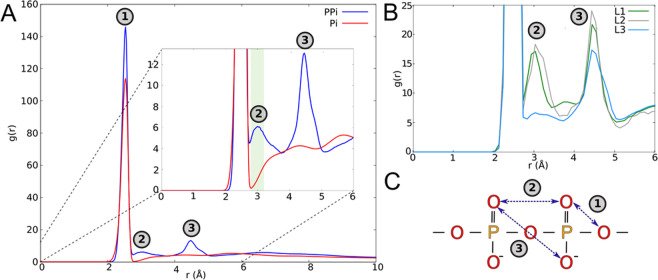
Oxygen–oxygen radial distribution function (gOO) for membrane-bound lipid II and phospholipids. **(A)** gOO for all oxygen atoms in PPi (*blue*) and Pi (*red*) groups reveals three maxima (*numbered*). *Inset*: zoomed-in fragment of gOO; highlighted with *green background* maximum #2 at 3.0 Å is unique for PPi (see also Fig. S5). **(B)** gOO for solvent-accessible oxygen atoms in different conformations of lipid II: L1 (*green*), L2 (*grey*), and L3 (*light blue*) (see Fig. 3). **(C)** Schematic representation of characteristic distances between oxygen atoms in PPi corresponding to gOO maxima (#1, #2, #3) in (A) and (B).

Since PPi plays crucial role in lipid II recognition^14,19,29^, it has to offer a unique H-bond acceptor pattern to facilitate its capture by extracellular lantibiotics in a rather chemically similar environment. To discover what is special about pyrophosphate’s oxygen atoms in comparison to phospholipids’ phosphates, we analyzed the oxygen–oxygen radial distribution functions (gOO) for both PPi moiety in lipid II and Pi groups in phospholipids (Fig. 4A). The first maximum (at 2.5 Å) is common for both functions and corresponds to adjacent oxygen atoms bound to the same phosphorus atom — whether from PPi or Pi (Fig. 4C; #1). Importantly, PPi gOO distribution exhibits a distinct second maximum at 3.0 Å (while this is not the case for phospholipids), corresponding to the oxygen atoms bound to the neighboring phosphorus atoms inside PPi (Fig. 4A, *blue* plot and *inset*; #2), which is probably responsible for lipid II recognition and hereinafter referred to as pyrophosphate (or O–O) pharmacophore.

Furthermore, not only the unique O–O distance is essential for lipid II recognition by lantibiotics, but also simultaneous exposition of these atoms to the membrane surface. To verify this assertion we derived analogous gOO function, taking into account only *solvent-accessible* oxygen atoms of PPi. Its analysis shows that only L1 and L2 states, but not L3, exhibited the required O–O pharmacophore conformation (Fig. 4B).

The gOO function for membrane lipids reaches close-to-zero minimum near r = 2.9 Å, but growths rapidly in the vicinity of 3.0 Å. This growth may be due to a rare but long-lived close approaches of Pi groups (competing with O–O pharmacophore) or their frequent but unstable rearrangements. By calculating pairwise distances between oxygen atoms of lipids, we elucidated that Pi groups in POPG/POPE bilayer do frequently approach to this distance, and only a few of them may persist for a relatively long time (≈20 ns). Such configurations represent mostly cation-binding sites, where Na^+^ ions bridge the neighboring lipids together. However, these oxygen pairs seem to be poor PPi competitors for nisin binding, as the presence of a cation tends to repel the positively charged nisin. In the reference zwitterionic POPC membrane, lipids’ Pi groups manifest solvent-accessible O–O pairs at 3.0 Å distance for no longer than 10 ns (Fig. S6), further diminishing this effect.

Importantly, the specific O–O pharmacophore in lipid II is stable only in the lipid bilayer mimicking bacterial membrane. The lifetimes of solvent-accessible oxygen atoms of PPi at 3.0 ± 0.1 Å distance in POPG/POPE bilayer may achieve 140 ns (Fig. S6), being a vivid peculiarity of lipid II molecule, while in the reference POPC bilayer it does not exceed 20 ns. Thus, the PPi group of lipid II provides a unique arrangement of H-bond acceptors in the bacterial-like membrane. We suggest that this is a principal feature, which makes lipid II a specific target for the membrane-active antibiotics that recognize its PPi group.

### Conformational dynamics of nisin and its recognition module in water/DMSO

MD simulations of intrinsically disordered peptides such as nisin are often complicated by a sampling bias. In order to accelerate computations and decrease the dimensions of the system we decided to focus on a truncated nisin analogue — its recognition module, containing just rings A and B. This N-terminal fragment, further referred to as nisin_1–11_, seems to be an optimal choice for MD simulations, as it is the minimal nisin analogue, retaining antimicrobial activity^28,39^ (most probably by depleting lipid II pool in the bacterial membrane). To confirm the conformational space similarity of the recognition module on its own and as a part of the whole nisin, we performed two series of MD simulations (see *Methods*).

In the first one, full-length nisin in aqueous solution was simulated. To get the first insight into its conformational lability, the RMSF was calculated for each residue. Most flexible regions turned out to be outside the macrocycles, i.e. N- and C-termini (RMSF ≈ 6 Å*vs*. 1.5–3 Å in lanthionine rings). As expected, no evidence of regular secondary structure elements, i.e. α-helices or β-sheets, was found. Due to the relatively extensive and chaotic intramolecular hydrogen-bonding network (11 ± 2 bonds on average), the molecule tends to adopt globular rather than extended conformation. However, no interaction between two N-terminal rings and the C-terminal region was detected. Rings A and B remained solvent-accessible (50–60% and 40–60%, respectively) during the overall simulation time, and thus were capable of forming intermolecular contacts.

**Figure 5:**
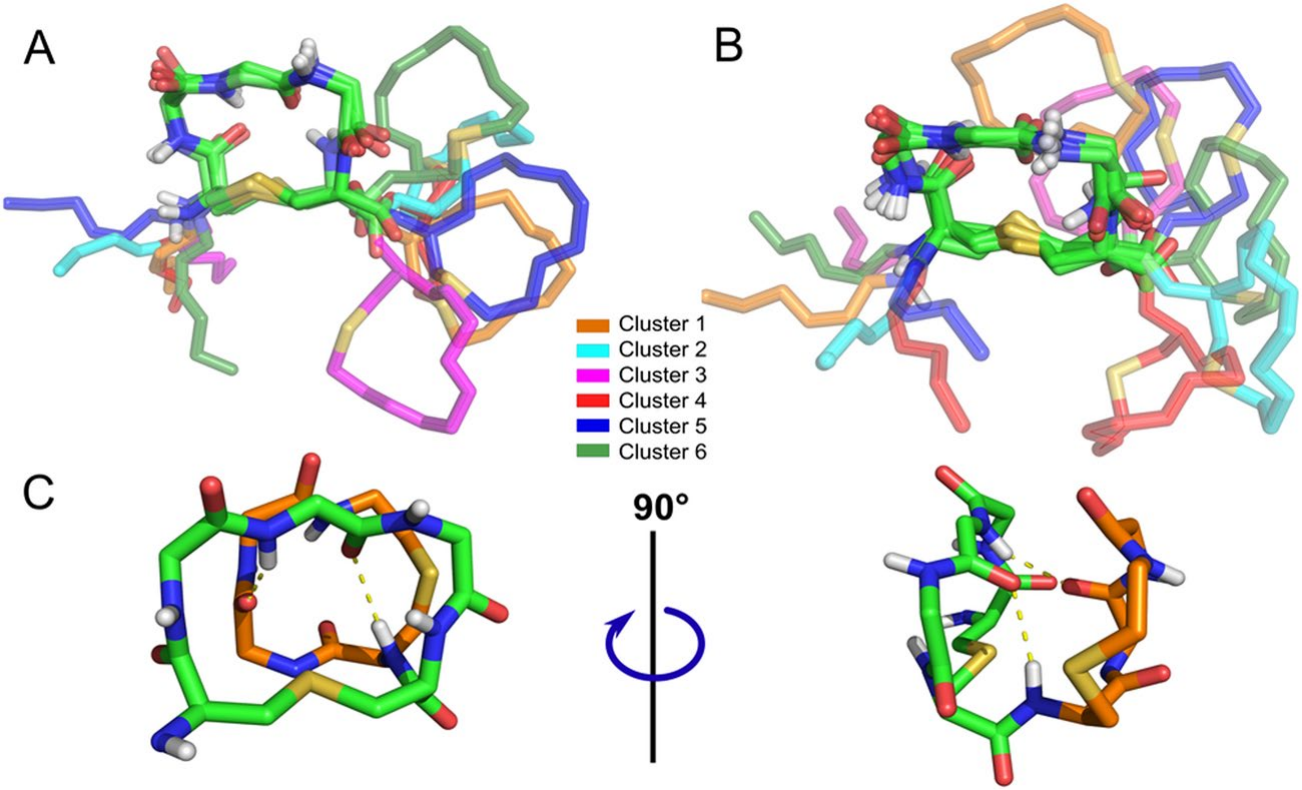
Characteristic conformations of nisin_1–11_ in water. **(A)**Representative structures of six states NF1–6 for rings A and B in full-length nisin. **(B)** Representative structures of six nisin_1–11_ states N1–6. The structures are superimposed the by ring A backbone (*green sticks*), while the ring B and the N-terminus (*colored tube* + *backbone*) positions significantly vary. Clusters of conformers are numbered as their population decreases. **(C)** Middle structure from the cluster N1. Spatial proximity of rings A and B induces rearrangement of H-bonds network (*yellow dotted lines*); four amide groups of the ring A co-orient to the probable O–O pharmacophore position.

Clustering by residues 1–11 backbone revealed six conformations (NF1–6; Fig. 5A). The N-terminus is highly flexible and predominantly sticks out into the solvent rather than interacts with the rest of the molecule. Rings A and B are connected by regular peptide bond between Ala7 and Abu8 residues. Thus, the relative position of the rings is determined by free rotation around this “hinge” and can be described in terms of torsion angles ψ_7_ and φ_8_. Ring B appears to have two preferred positions, described by φ_8_ = 70 ± 15°; 114 ± 13°, while ring A adopts at least four favored positions with ψ_7_ = −39 ± 23°; 25 ± 11°; 110 ± 17°; and 148 ± 13°.

Ring A backbone structure is exceedingly stable with notable fluctuations in thioether bridge region only. Two intra-macrocycle H-bonds between oxygen atoms of Ala3, Dha5 and the amide groups of Dha5, Ala7, respectively, contribute towards stabilization of the preferred state. Consequently, the direction of H-bond donors, i.e. amide NH groups, is well-defined. These findings are in agreement with the NMR data^10^. Despite being smaller and containing proline, ring B exhibits higher backbone flexibility. Residues Abu8 and Gly10 were found to rapidly switch orientation of their CO and NH groups with respect to the ring plane. The equivalent experiment in DMSO identified that nisin maintained an extended (not globular) structure in an aprotic solvent. However, this does not affect the ensemble of the lipid II recognition site.

In a second MD series we proceed with a simplified model system — recognition module of nisin (nisin_1–11_), that was found to adopt essentially the same ensemble of six conformations (N1–6) as the full-length peptide, but with different population of the states (Fig. 5B). The most remarkable variation is observed within cluster 1, where the more cramped rings position (Fig. 5C) and H-bond network rearrangement was registered. The newly-formed inter-ring H-bonds between Dha5 and Abu8 and Pro9 sculpt ring A backbone such that its four NH groups are co-directed at one side in respect to the ring plane, forming the probable competent conformation (N1) for recognition of the lipid II O–O pharmacophore (Fig. 5C). Characteristic values of ψ_7_ are −42 ± 13°; 25 ± 14°; 100 ± 13°; 145 ± 17°; and values of φ_8_ are −46 ± 10°; 121 ± 14°.

It is important to note that recently published data on NMR ensemble of nisin_1–12_ revealed the very similar set of 6 conformations in solution, within which the N1 state described above was observed^40^. Thus, nisin_1–11_ represents a good model system of full nisin, since the C-terminal membrane-active module does not participate in lipid II recognition, but instead may contribute to decoy conformations, and, more importantly, recognition module structure and dynamics are preserved in both cases.

### PPi analogue induces conformational switch in nisin_1–11_ upon binding in water

**Figure 6:**
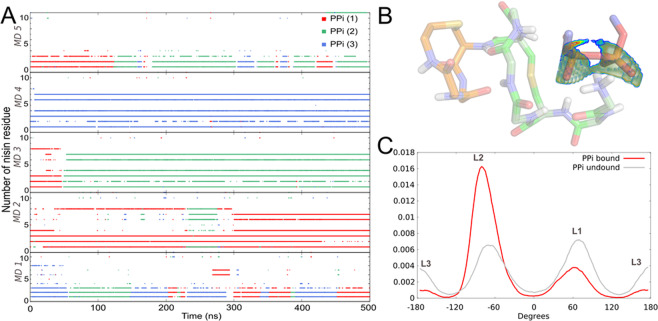
Spontaneous formation of nisin_1–11_/DMPPi complexes in MD. **(A)**Hydrogen bonds map between nisin_1–11_ and DMPPi ions. Each dot indicates H-bond between different DMPPi ions (*different colors*) and particular nisin_1–11_ residue (along the *vertical axis*) at a given MD time (along the *horizontal axis*). Experiment was repeated five times, as shown by five graphs one under the other. For most of MD time, one DMPPi ion is trapped by nisin_1–11_. Strong complexes formed in MD 2 and 3 correspond to the relevant (N1) state of nisin_1–11_. **(B)** Snapshot of the structure of stable nisin_1–11_/DMPPi complex in water. The *volume surface* indicates predicted density-colored positions of the O–O pharmacophore, interacting most favourably with a given nisin_1–11_ conformation (according to E_OO_ criterion, see text). The oxygen atoms of PPi moiety observed in MD simulation inscribe well into this predicted density (see Video S1). **(C)** Distribution of OPPO torsion angle in DMPPi over MD trajectories. Note significant change of angle values for bound molecule (*red*) and proportional occurrence of three states for molecule in solution (*grey*).

We investigated mutual adaptation of nisin_1–11_ and a “minimal” lipid II analogue that contains a PPi group — dimethyl pyrophosphate (DMPPi) — upon their interaction in aqueous solution. Nisin_1–11_/DMPPi complexes formed spontaneously and remained more or less stable in all five MD trajectories. DMPPi binding site is located within the first seven nisin residues, i.e. ring A and the N-terminal Ile1 and Dhb2. DMPPi ions succeeded each other in the binding site, unless nisin_1–11_ adopted conformation similar to N1 (Fig. 5C). DMPPi binding stabilized this conformation: the structure and relative position of rings A and B remained unchanged for >450 ns *vs*. maximum 100 ns for isolated nisin_1–11_. In addition, the number of intermolecular H-bonds for this complex is 4–6 compared to mere 3–4 for other nisin_1–11_ conformations (Fig. 6A). Residue Abu8, which interacts with PPi in the NMR structure^29^, did so extremely rare in our MD experiment.

In the N1-like DMPPi bound state, N-terminal and ring A amide groups of nisin_1–11_ orient towards a common center, forming a pool of H-bond donors (Fig. 6B). Interestingly, this is very similar to the recently resolved X-ray structure of another lipid II’s PPi binder — depsipeptide teixobactin analogue, which coordinated chloride anion by five NH groups^41^. Moreover, the similarity of nisin and teixobactin binding motif to lipid II was revealed in a computer experiment^42^.

PPi is also affected by the peptide presence. Floating freely in the solution, DMPPi reproduces the conformational ensemble of PPi within the membrane-bound lipid II (L1–L3; Fig. 3). In the course of MD simulations, there is fast conformational exchange between these states with nearly equal probability of L1-L3 states (Fig. 6C). Binding to nisin_1–11_ confines DMPPi conformational ensemble, almost excluding the L3 conformation and significantly increasing probability density of L2 state (OPPO ≈ −60°). Thus, we receive further support for the conclusion that only two (L1 and L2) out of three states of the PPi group are optimal for the interaction with nisin.

The stable complex identified in our simulations (Fig. 6B, Video S1) has not been described previously and is different from the NMR structure^29^, which may reflect environmental differences, since the experiment was conducted in DMSO solution, instead of membrane. To elucidate this supposition, we conducted a series of five independent MD simulations of the same system in DMSO. Indeed, the conformational space of nisin_1–11_ was affected by bipolar medium: N1 conformation did not appear in any trajectory, and the observed complexes employed the N-terminal amide groups only (residues 1–4). Nisin_1–11_ conformation similar to that in the NMR-model of the complex (PDB ID: 1WCO) occurs significantly more frequently in DMSO as compared to water solution (55 and 13% MD-frames, respectively) (Fig. S7).

Analysis of the spontaneously formed complexes in water revealed the medium-guided induced-fit mechanism. We assume that the pharmacophore ensuring PPi recognition is characterized by co-directional disposition of NH vectors pointing to the pair of target PPi oxygen atoms in lipid II. To confirm this hypothesis, we developed an energy-based scoring function, further referred to as “energy of the pyrophosphate O–O pharmacophore” (*E*_OO_).

### The “energy of the pyrophosphate O–O pharmacophore” function detects complex-forming conformations of nisin

The essence of the proposed approach is to identify complex-forming conformations of isolated molecules of nisin_1–11_ from its conformational ensemble. For this purpose, we have developed an independent energy-based criterion based on the O–O pharmacophore feature described above.

It is noteworthy that the sampling problem seriously affects all energy-based methods. Even in case of rather small yet highly-flexible molecules, such as nisin and lipid II, we have to study simplified systems. To discover an optimal DMPPi binding mode for a given conformation of nisin_1–11_, we further simplify DMPPi molecule to a pair of atoms O–O, delimited by a characteristic distance of ≈3 Å – as follows from the gOO analysis (Fig. 4A). For such a ultimately simplified system, a thorough conformational scan becomes possible, thus we define energy of the pyrophosphate O–O pharmacophore (*E*_OO_) as non-bonding interaction energy of this atom pair with a given nisin_1–11_ conformation (see *Methods* for details).

The calculated 3D distribution of the *E*_OO_ for N1 state of nisin_1–11_ contoured by iso-potential surfaces is shown in Fig. 6B; it reveals the optimal position of the probe O–O atoms pair that interacts with the recognition module of nisin. Thus, we can estimate which amide groups of the peptide are most likely to interact with the model target, and what is the best (with low *E*_OO_) location of the probe with respect to the peptide. One should bear in mind that *E*_OO_ is not the true binding free energy, but rather a criterion for comparing different peptide conformations and identifying the complex-forming ones, as well as the optimal sites for binding of O–O pair.

**Figure 7:**
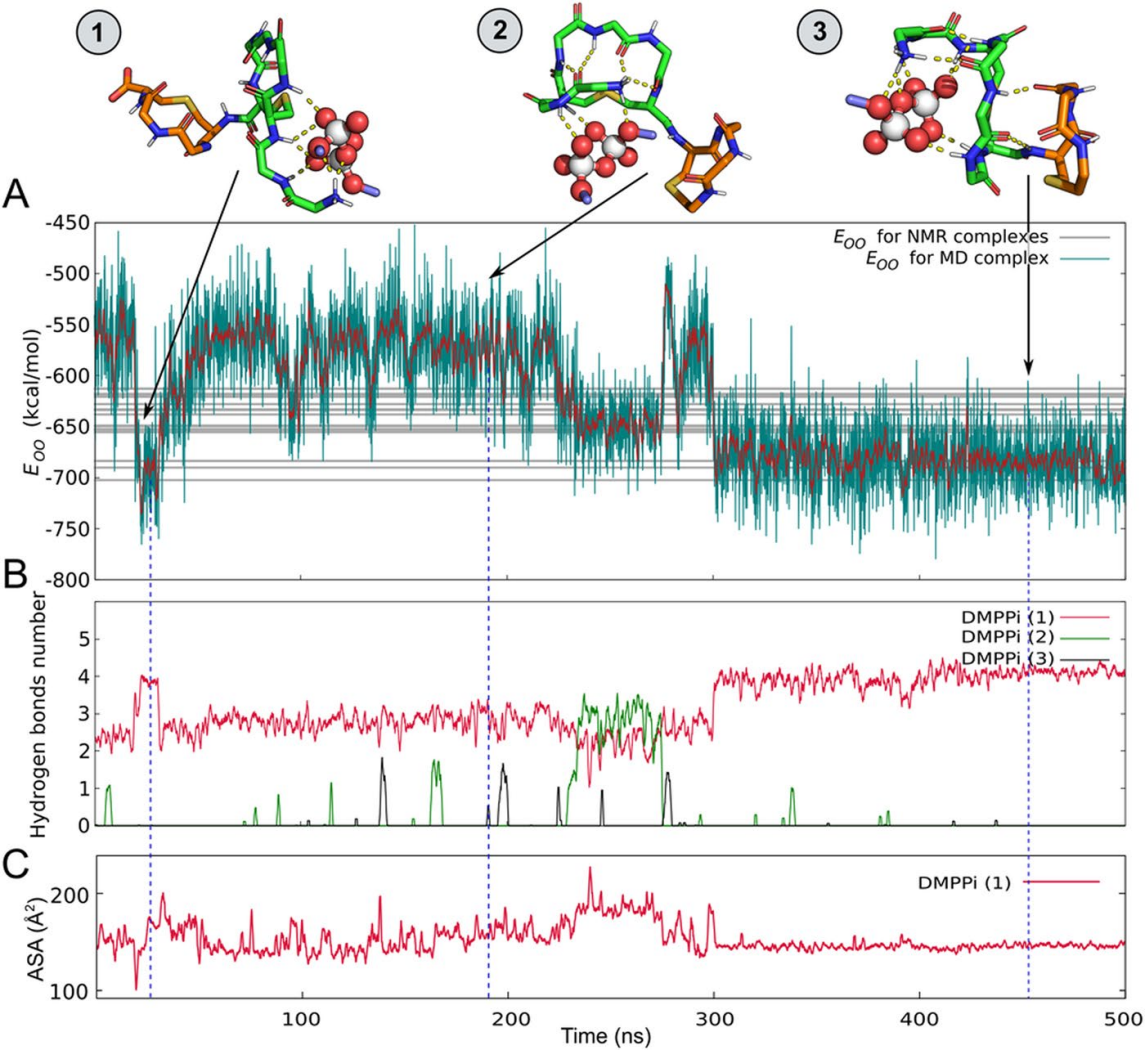
Identification of complex-forming conformations of nisin from the MD ensemble using the “Energy of the pyrophosphate pharmacophore” (E_OO_) approach. This is an example of MD-2 from Fig. 6A. **(A)** E_OO_ profile for nisin_1–11_/DMPPi complexes, spontaneously forming in water solution. E_OO_ (instant and time-averaged (1 ns window) values are shown with *green* and *red lines*, respectively) as a function of MD time illustrates the complex formation. Note that E_OO_ describes solely nisin_1–11_ conformation, not the DMPPi interaction energy. *Grey lines* correspond to calculated E_OO_ values for 20 NMR structures of nisin/lipid II analogue complex in DMSO[29]. The relevant complex snapshots are shown at the top. Carbons of rings A and B of nisin are coloured green and orange, respectively. DMPPi is shown with spheres and purple sticks. **(B)** The time-averaged (0.1 ns window) number of H-bonds between nisin_1–11_ and DMPPi ions (*individually coloured*), which exhibits anticorrelation with E_OO_ values (R = −0.62). **(C)** The time-averaged (1 ns window) solvent accessible surface area (ASA) of the nisin_1–11_ bound to DMPPi during the simulation.

Firstly, to validate the method’s efficacy and establish the range of the optimal “probe” energies, we computed *E*_OO_ for nisin conformations obtained from MD trajectory of nisin_1–11_/DMPPi complex. The predicted position of the probe oxygen atoms pair revealed insignificant deviations from the real one in the observed complexes (Fig. 6B). During MD run, *E*_OO_ decreased from −500 to −750 kcal/mol (Fig. 7A). This illustrates nisin_1–11_ conformational changes that accompany formation of the complex *via* the number of intermolecular H-bonds (Fig. 7B).

We found two distinct low-energy (*E*_OO_) states of nisin_1–11_ in water, which both bind DMPPi via four H-bonds: “transient” complex at 20–35 ns (Fig. 7, complex #1; *E*_OO_ ≈ −688 kcal/mol) and “stable” complex with nisin_1–11_ during the last 200 ns (Fig. 7, complex #3; *E*_OO_ ≈ −682 kcal/mol). Despite similar *E*_OO_ values, complexes #1 and 3 differ greatly in the backbone structure of nisin_1–11_ and relative position of DMPPi. In complex #1, DMPPi is exposed to solvent and binds to four N-terminal residues, while the ring A and B are almost not involved in complex formation. Oppositely, in complex #3, nisin_1–11_ adopts N1 conformation, and DMPPi is located in a cavity formed by the ring A and the N-terminus, with its accessible surface decreasing (Fig. 7C), and the ring B stabilizes this conformation *via* two H-bonds.

In the transient complex (#2), nisin_1–11_ traps DMPPi by four N-terminal residues, rather than by the ring A as it does in stable complexes (compare complexes #1 and 3 in Fig. 7). Interestingly, the same transient complex structure (#1) was found in MD trajectories in DMSO solution with even lower *E*_OO_ ≈ −761 kcal/mol and lifetime over 250 ns (Fig. S8, complex #2). We suppose that such a drop in *E*_OO_ is due to unavailability of alternative hydrogen bond acceptors in DMSO and resulting decrease in flexibility of nisin’s backbone amide groups.

*E*_OO_ for the set of experimental NMR structures of nisin/lipid II analogue in DMSO equals to −643 ± 25 kcal/mol with the energy of representative (conformer 1) structure being −629 kcal/mol (*grey lines* in Fig. 7A). The stable complex from MD has *E*_OO_ ≈ −683 kcal/mol (last 200 ns in Fig. 7, complex #3) competing with NMR complexes. The calculated *E*_OO_ values for different states are given in Table 1. These values were used as a reference to identify the complex-forming states in MD trajectories of isolated nisin.

**Table 2:** Computations implemented in this study.

System composition	MD length (ns)	Number of trajectories
*Lipid II in membrane environment*		
Lipid II/POPG_186_/POPE_66_/Water_13701_/Na^+^_189_	1000	3
Lipid II/POPC_286_/Water_10502_/Na^+^_3_	1000	1
*Full-length nisin in solution*		
Nisin/Water_15862_/Cl^−^_3_	100	4
	1000	1
Nisin/DMSO_14680_/Cl^−^_3_	100	4
*Nisin*_*1–11*_ *with or without DMPPi in solution*		
Nisin_1–11_/DMPPi_3_/Water_5686_/Na^+^_6_	500	5
Nisin_1–11_/DMPPi_3_/DMSO_10992_/Na^+^_6_	500	5
Nisin_1–11_/Water_5740_	400	5
Nisin_1–11_/DMSO_6576_	400	5
*Nisin/lipid II complex in bacterial membrane*		
Nisin_1–11_/Lipid II/POPG_186_/POPE_66_/Water_13701_/Na^+^_189_	1000	3

To assess the fraction of the complex-forming conformations in free nisin_1–11_, we calculated *E*_OO_ for nisin_1–11_ trajectory without DMPPi in both solvents (Fig. S9). In more than 60% of MD-states, isolated nisin_1–11_ molecule may potentially form a low-energy complex with PPi (*E*_OO_ = −586 ± 32 kcal/mol). *E*_OO_ values sufficient for stable complex formation (<683 kcal/mol — the *E*_OO_ value of complex #3 in Fig. 7) appear very rarely — at 0.4% of MD time, mostly corresponding to the complex-forming state of nisin_1–11_ (conformation N1).

Thus, whether we explored nisin_1–11_ conformations in the presence or absence of the target, the highest binding capacity is observed for the complex-forming state, which distinguishes itself by a pronounced pool of H-bond donors that can bind pyrophosphate.

### Probable nisin/lipid II complex in model bacterial membrane

Nisin as an extracellular agent has to specifically recognize lipid II on the bilayer surface. We suggest that both partners are flexible in their parent environments, sometimes adopting competent conformations for mutual recognition: N1 for nisin and L1 or L2 for lipid II (see Fig. S10). Moreover, upon interaction, the populations of competent conformations increase, exhibiting an induced-fit mechanism.

**Figure 8:**
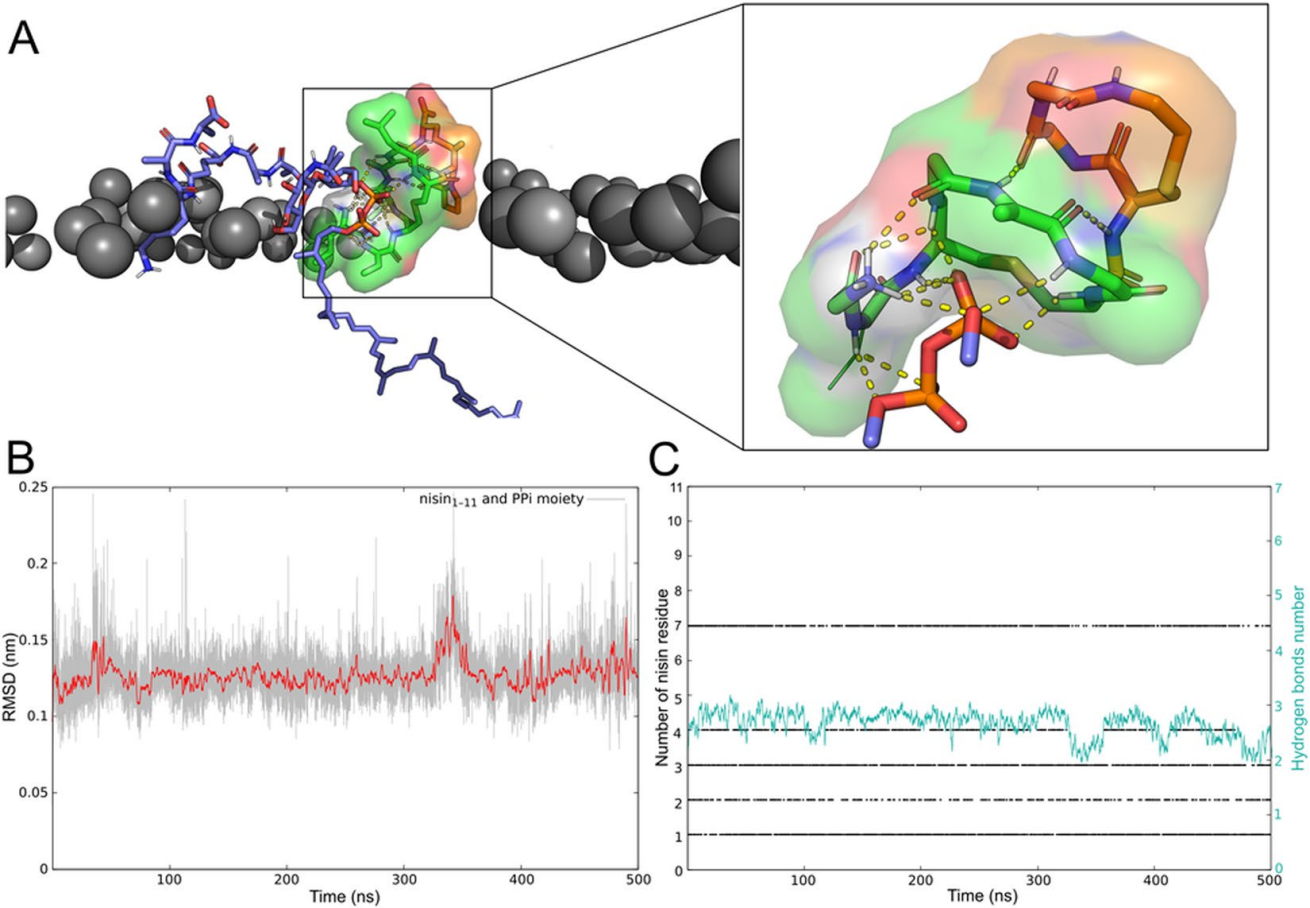
Model of nisin_1–11_/lipid II complex in bacterial membrane and its stability in MD simulation. **(A)** Binding mode of nisin_1–11_ with respect to the bilayer surface and lipid II’s PPi group. Rings A and B of nisin are represented with *green* and *orange sticks*, respectively; lipid II is shown with* purple sticks*. Phosphorus atoms of lipids are shown as *grey spheres*. H-bonds are shown with *yellow dotted lines*. **(B)** Structural stability of the complex. Root-mean-square deviation (RMSD) values relative to the initial structure calculated for nisin_1–11_ and PPi moiety of lipid II during the simulation time (*grey*) and averaged by 0.1 ns (*red*). **(C)** Hydrogen bonds map. Black dots indicate H-bond between PPi moiety and certain nisin_1–11_ residue (along the *left vertical axis*) at a given MD time (along the *horizontal axis*). Nisin_1–11_ binds to the lipid II’s PPi group in a similar way to that of DMPPi in solution. The average number (0.1 ns time window) of H-bonds is shown with *green line* (*right axis*).

Based on the found probable competent conformations of both molecules, we constructed four possible models of the complex in the membrane. This was done via alignment of lipid II’s states L1 or L2 to DMPPi (in N1-DMPPi complex) each in two ways due to the DMPPi symmetry. And only one of N1-L1 was not sterically hindered (Fig. 8A). In the course of 500-ns MD simulation, the complex was stable (Fig. 8B), simultaneously forming 4–5 H-bonds (Fig. 8C). Moreover, H-bond pattern remained the same as in aqueous solution, excluding Leu6, which did not participate in binding.

## Discussion

Intermolecular recognition is the core phenomenon in molecular biology, driven mostly by weak interactions, such as hydrogen bonding and electrostatic effects. Similar to the induced-fit mechanism^43^ in enzymatic catalysis, the ligand and the receptor, reciprocally recognizing each other, may undergo conformational “fine-tuning”, especially if they are highly flexible. Lipid II is an agent of bacterial membranes, which consists of highly flexible undecaprenol tail and peptidoglycan head, connected by a “hinge” — PPi moiety, which appears to be an attractive chemically conserved target for AB action.

Our calculations show that only the gauche^+^ and gauche^−^ (OPPO torsion ≈ ±60°), but not the anti-conformation (≈180°) of lipid II are competent for capture by nisin due to accessibility of the O–O recognition pharmacophore to the solvent. The lipid environment strongly modulates this pharmacophore: when embedded into the native-like (i.e., mimicking bacteria) POPG/POPE membrane, lipid II slows down the conformational exchange of PPi, as compared to the reference POPC bilayer. This results in significantly longer pharmacophore lifetimes on the bilayer surface. Pi groups of neighboring phospholipids may also form intermolecular O–O pairs at ≈3 Å separation — similar to the O–O pharmacophore of lipid II, but they are either short-lived (in POPC) or Na^+^-associated, thus hampering their interaction with cationic antimicrobial peptides (in POPG/POPE). That is why lipid II is the only molecule in the studied model membranes that provides definite high-affinity interaction site for nisin and related compounds.

The conformation of nisin_1–11_, that can form a complex with lipid II (Fig. 6) is characterized by several backbone NH groups of the N-terminus and the ring A, co-oriented towards the O–O lipid II pharmacophore. Ring B plays crucial role in stabilization of this state forming two H-bonds between oxygen atoms of Ala3, Dha5 and the amide hydrogens of Dha5, Ala7, respectively. The most important finding is that nisin_1–11_ competent conformation emerges in water only and not in DMSO, where the spatial structure of the nisin/lipid II complex was solved by NMR^29^. This provides evidence that the nisin/lipid II recognition mode defined by the latter structure is not unique. Recently published NMR data on the conformational ensemble of nisin_1-12_ in an aqueous solution^40^ confirmed the existence of the predicted N1 conformation, which to some extent validates the proposed computer models.

It is important to note that for the first time the pattern of co-oriented NH groups was revealed in the NMR structure^29^ (Fig. 1C). Next, recently resolved X-ray structure of teixobactin analogue capturing chloride anion^41^ demonstrated a very similar cavity fulfilled with amide NH groups that can bind a target. We suggest such pharmacophore structure to be conservative along ABs targeting the PPi moiety of lipid II. In this work, we developed an energy-like scoring function (*E*_OO_)to identify competent conformations of nisin recognition module from ensemble of decoys, naturally emerging from high flexibility of nisin. With this approach, reflecting the main idea that the recognition module should be competent to capture at least two oxygen atoms delimited by a very specific distance of ≈3 Å, we were able to extract from MD trajectories the “lowest-energy” states with presumably the highest potency for interaction. We suggest that our method or its analogues may be useful for further studies of lipid II-capturing agents and rational design of novel compounds that may stand for prototypes of novel classes of ABs. Our ongoing research will probe other known lipid II recognizing structures to match the pharmacophore hypothesis elaborated in this work.

Since 2004, the only picture of lipid II recognized by lantibiotics is the well-known NMR structure of the complex of nisin with shortened analogue of lipid II in DMSO^29^. This structure revealed the main principle of the recognition — co-direction of several nisin backbone NH groups into a common binding site — but not made it clear what is so special about PPi, that it represents specific nisin target in a “sea” of surrounding phospholipids. Moreover, DMSO is an artificial medium, that certainly affects native nisin conformation and might alter the complex structure as compared to aqueous solution and, even more so, in the heterogeneous membrane environment.

In this work, we propose a probable structure of nisin recognition module (nisin_1–11_) with lipid II in its natural environment (Fig. 8A) — model bacterial membrane. Lipid composition POPG:POPE = 3:1 is a good mimic for the gram-positive bacteria membrane^38^, against which nisin has high potency^44^. Note that complex structure was determined from atomistic MD simulations of nisin_1–11_ with PPi analogue and not based upon the NMR structure^29^. We assembled nisin_1–11_/lipid II complex on the membrane surface using the conformations of individual molecules, and showed its stability for at least 500 ns. However, this model has to be completed towards the original nisin structure, and the simulation time should be increased.

### Nisin/lipid II recognition: an induced pharmacophore

It was shown that addition of lipid II to membrane-mimicking systems ≈1000-fold increases nisin affinity^14,18^. The reason is that lipid II is a highly specific target for nisin (and many other membrane-active antibiotics) action, and it can be compared to the Achilles heel of a bacterial membrane. The common vision of this process is that capturing lipid II by nisin’s N-terminal recognition module facilitates effect of the C-terminal membrane-active module, and at the same time withdraws lipid II from cell wall synthesis machinery, thus blocking the process.

But what is the mechanism of this recognition, given that lipid II seems to be “a drop in the ocean” of phospholipids and proteins^19^? By bringing together well-known mechanisms of AMP’s action^45–47^, our previous^35^ and current results, we propose the following scheme:

- Positively-charged AMP (nisin has net charge +3*e*) is electrostatically attracted to the negatively-charged surface of bacterial membranes (this feature differentiates them from zwitterionic eukaryotic membranes^48^);
- Most membrane-active peptides are amphiphilic. We hypothesized^35^ that lipid II induces the reciprocal amphiphilic pattern on the membrane surface. According to this hypothesis, a “hydrophobic atoll” that surrounds the head of lipid II may serve as a “landing terrain” for nisin and many other AMPs, converting the search from 3D space to 2D vicinity.
- Nisin adsorption at lipid II “atoll” brings two molecules together. “Flickering” competent conformations of both partners finally meet each other, initiating recognition, leading to the induced fit and, finally, to the formation of the stable complex.

Based on the available literature data, we can suppose that the stable complex between nisin’s N-terminal recognition module (nisin_1–11_) promotes two subsequent (and independent) events:

- Lipid II pool is depleted, and cell wall synthesis stops^14^;
- Nisin C-terminal membrane-active module disturbs membrane and forms the pore in the membrane^19^, thus dissipating membrane gradients and killing bacteria.

## Methods

### Molecular dynamics (MD) simulations

MD simulations were carried out in GROMACS package, version 5.1.2^49^ using Gromos96 43a2x force field, 2 fs integration time step and imposed 3D periodic boundary conditions. A 12 Å spherical cut-off function was used to truncate van der Waals interactions. Electrostatic effects were treated using the particle-mesh Ewald summation^50^ (real space cutoff 12 Å). The temperature and pressure of the systems were maintained by V-rescale coupling method^51^ at 315 K and Berendsen coupling method^52^ at 1 bar, respectively. MD simulations were performed using an improved version of lipid parameters introduced by Berger *et al*.^53^ and SPC water model. A certain number of solvent molecules were replaced by Na^+^ or Cl^−^ ions to make each system electrically neutral.

Prior to MD simulations, all systems were subjected to energy minimization (1000 conjugate gradients steps). Each system was subsequently heated to 315 K. Peptide, lipid II, lipids and solvent molecules were coupled separately. MD simulation of each system was repeated at least three times by random assigning initial velocities. All simulations made are listed in Table 2.

#### MD of lipid II in membranes

The membrane set up was performed using our in-house framework IMPULSE. Each bilayer leaflet was either a mixture of POPG:POPE lipids 3:1 for model bacterial membrane or pure POPC for reference zwitterionic (eukaryotic) bilayer. The bilayers were placed into rectangular boxes (typical size 83 × 83 × 106 Å^3^ and 92 × 92 × 88 Å^3^, respectively) and solvated with SPC water molecules (Table 2). Lipid II starting structure was taken from a simulation that has been published previously^35^. The molecule was inserted into the pre-equilibrated bilayers using IMPULSE utility, with overlapping phospholipids removed. Lipid II position in the bilayers was adjusted to match PPi group Z-coordinate with the Pi groups plane. The semi-isotropic pressure coupling in the bilayer plane and along the membrane normal was used in the simulation. MD-averaged value of the area per lipid in the presence of lipid II was 54.5 ± 0.3 Å^2^ and 59.8 ± 0.2 Å^2^ for POPG/POPE and POPC, respectively. The calculated membrane thickness was 40.2 ± 1.4 Å for heterogeneous POPG/POPE bilayer and 39.2 ± 1.2 Å for POPC.

#### MD of nisin in presence and absence of DMPPi

The set of five independent MD simulations both in water and in DMSO was performed for the following molecules: full-length nisin; nisin_1–11_; nisin_1–11_ with DMPPi (Table 2). Starting structure of the full-length nisin was retrieved from the PDB entry 1WCO (conformer 1). Initial coordinates for the nisin_1–11_ simulations were extracted from MD-equilibrated states of the full-length molecule. All systems were solvated in cubic water or DMSO boxes. To investigate mutual adaptation of the peptide and its target, three DMPPi molecules were randomly placed in a box with a minimum distance to the peptide of 5 Å.

#### MD of nisin/lipid II complex in the bilayer

The nisin_1–11_/lipid II complex in the POPG:POPE 3:1 membrane was assembled by aligning the stable complex structure from the solvent simulation (previous paragraph) with competent lipid II conformations (L1 and L2). Both conformers were selected so that PPi dihedral angle OPPO matched (62.5° and −63.8°). We constructed four possible models of the complex in the membrane, due to the symmetry of DMPPi, which doubled N1-L1 and N1-L2 combinations count. Single complex model lacked sterical overlap, i.e. there were no POPG and/or POPE lipid head groups intersecting the nisin_1–11_. The system was solvated with water molecules. During equilibration, distance restraints were applied to the peptide backbone, heavy atoms of lipid II head group and PPi. Long-term MD simulations were performed without restraints.

### Analysis of MD trajectories

The computed MD trajectories were analyzed using original GROMACS and custom IMPULSE utilities. Dynamic behavior and stability of the studied systems were assessed by RMSD, RMSF, dihedral angles, solvent accessible surface area (SASA) and H-bond profile calculations, using GROMACS built-in tools. Preferred conformations of nisin’s rings A and B were found using *cluster* module from the GROMACS package, where backbone atoms of residues 1–11 were superimposed (for data on clustering reproducibility see Table S1). A cut-off of 2.2 Å was applied, and the largest clusters (>1%) were extracted. Pairwise oxygen–oxygen radial distribution functions (gOO(r)) were calculated using *rdf* utility of the Gromacs package. The gOO(r) function of oxygen atoms exposed on the bilayer surface was calculated using the IMPULSE suite. Stability of the complex structure in the membrane was estimated by RMSD calculation of the atoms coordinates from the starting conformation. Only the subset of atoms corresponding to the model system in solution, i.e. backbone of nisin_1–11_ and pyrophosphate group of lipid II were included in the RMSD calculation.

### “Energy of the pyrophosphate O–O pharmacophore” (E_OO_) calculation

In-house *E*_OO_ score function was developed to explore the capacity of nisin_1–11_ to bind the target – a pair of oxygen atoms at a given distance of 2.75–3.25 Å, which is the “core” of the PPi pharmacophore. Nisin_1–11_ was placed into a rectangular box with 10 Å margins. A uniform cubic grid with a step of 0.2 Å was generated inside the box. At the first stage, a single probe oxygen atom (of OP Gromos type – oxygen in phosphate) was placed in every grid point, and the energy of nonbonded interactions (both Coulomb and Lennard-Jones) between probe atom and nisin fragment was calculated without any cutoff and switch/shift parameters. Next, the calculated energy grid map was analyzed to determine the following values:

- Positions of two grid points separated by a given distance (2.75–3.25 Å, unless otherwise specified) with a minimal total energy (i.e. sum of two energies). Thus, location of the second oxygen atom was selected to form the probe O–O pair;
- The minimal energy of the probe atoms pair (*E*_OO_);
- Positions of all pairs of grid points separated by the given distance and with the total energy less than *E*_OO_ + 10kT (T = 315 K).

The latter distribution was visualized by PyMol volume map option (Fig. 6B).

## Supplementary information

Supplementary information.

## Data Availability

Structure of the complex in the model bacterial membrane along with molecular topologies are deposited in the Zenodo archive (10.5281/zenodo.3572677).
