# Supplementary material for: Environmental and dynamic effects explain how nisin captures membrane-bound lipid II
Source: Sci Rep. 2020 Jun 1;10:8821. doi: 10.1038/s41598-020-65522-y (PMC7264305; doi:10.1038/s41598-020-65522-y)
Supplement: Supplementary file 1 — Supplementary information. [file 41598_2020_65522_MOESM1_ESM.pdf]

# Supplementary information

# How nisin captures membrane-bound lipid II: environmental and dynamic effects

I. S. Panina,[1,2] N. A. Krylov,[1,2] D. E. Nolde,[1,2] R. G. Efremov,[1,2,3]* A. O. Chugunov[1,2,3]

[1] Shemyakin-Ovchinnikov Institute of Bioorganic Chemistry, Russian Academy of Sciences, 16/10 Miklukho-Maklaya St., Moscow, 117997, Russia.
[2] National Research University Higher School of Economics, Moscow, 101000, Russia.
[3] Moscow Institute of Physics and Technology (State University), Dolgoprudny, 141701, Moscow Oblast, Russia.
*Corresponding author. Email: r-efremov@yandex.ru.

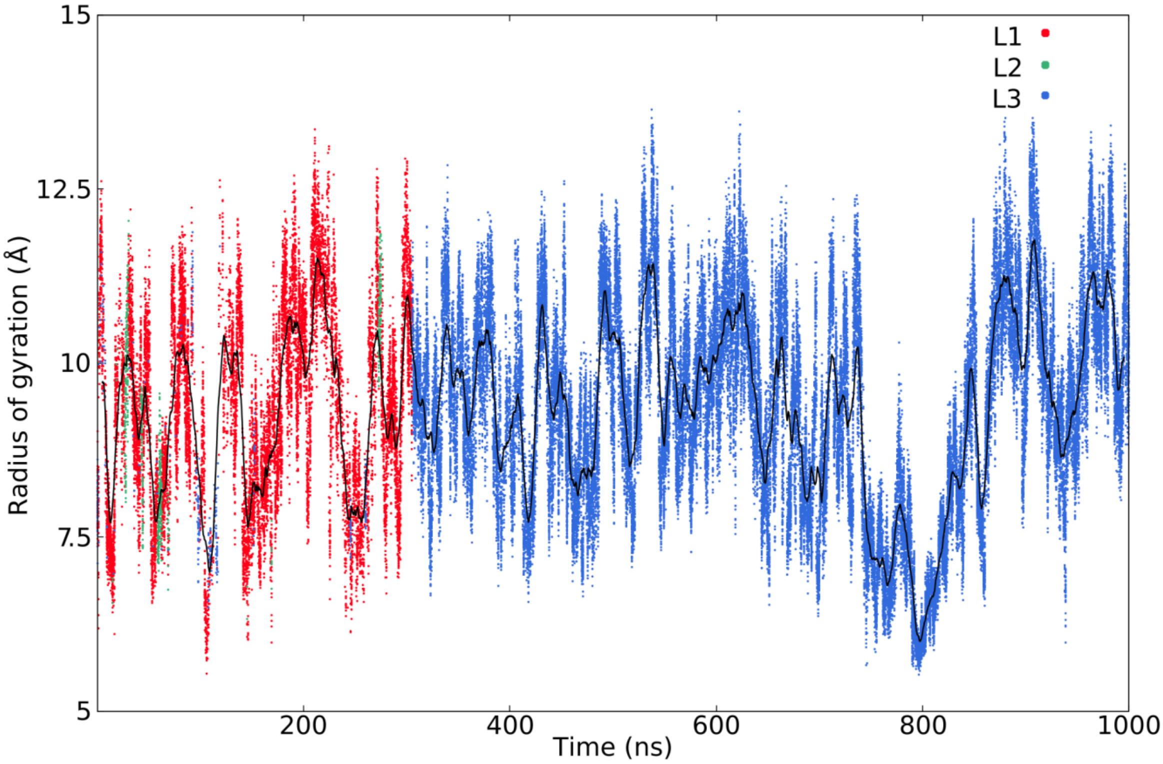


**Supplementary Figure S1. Lipid II's tail radius of gyration in the course of MD simulation.** Different colors correspond to three lipid II states: L1 (*red*), L2 (*green*) and L3 (*blue*) determined by OPPO dihedral value (see Fig. 3 and Fig. S3). Conformation of PPi group does not affect the tail mobility.

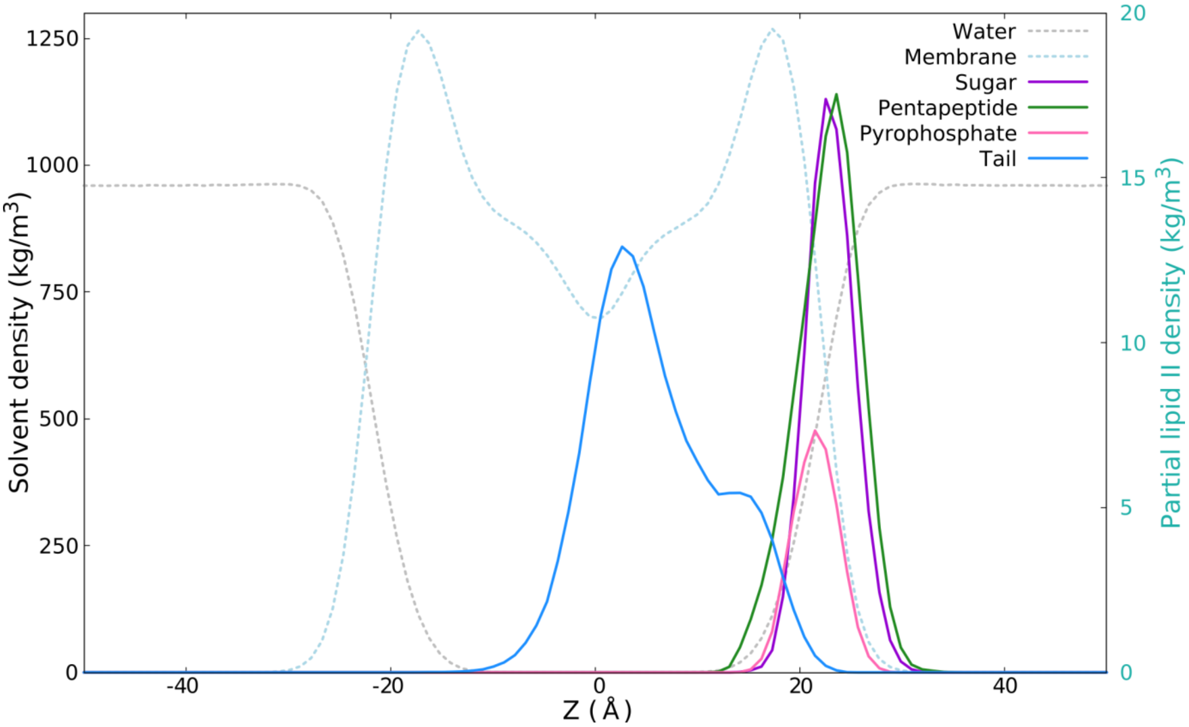


**Supplementary Figure S2. The average density profiles of various lipid II groups (*right axis*) and solvent (*left axis*) as a function of the distance from the bilayer center (Z).** Lipid II density is shown for the following groups: sugar residues (*violet*), pentapeptide (*green*), PPi (*pink*), tail (*blue*).

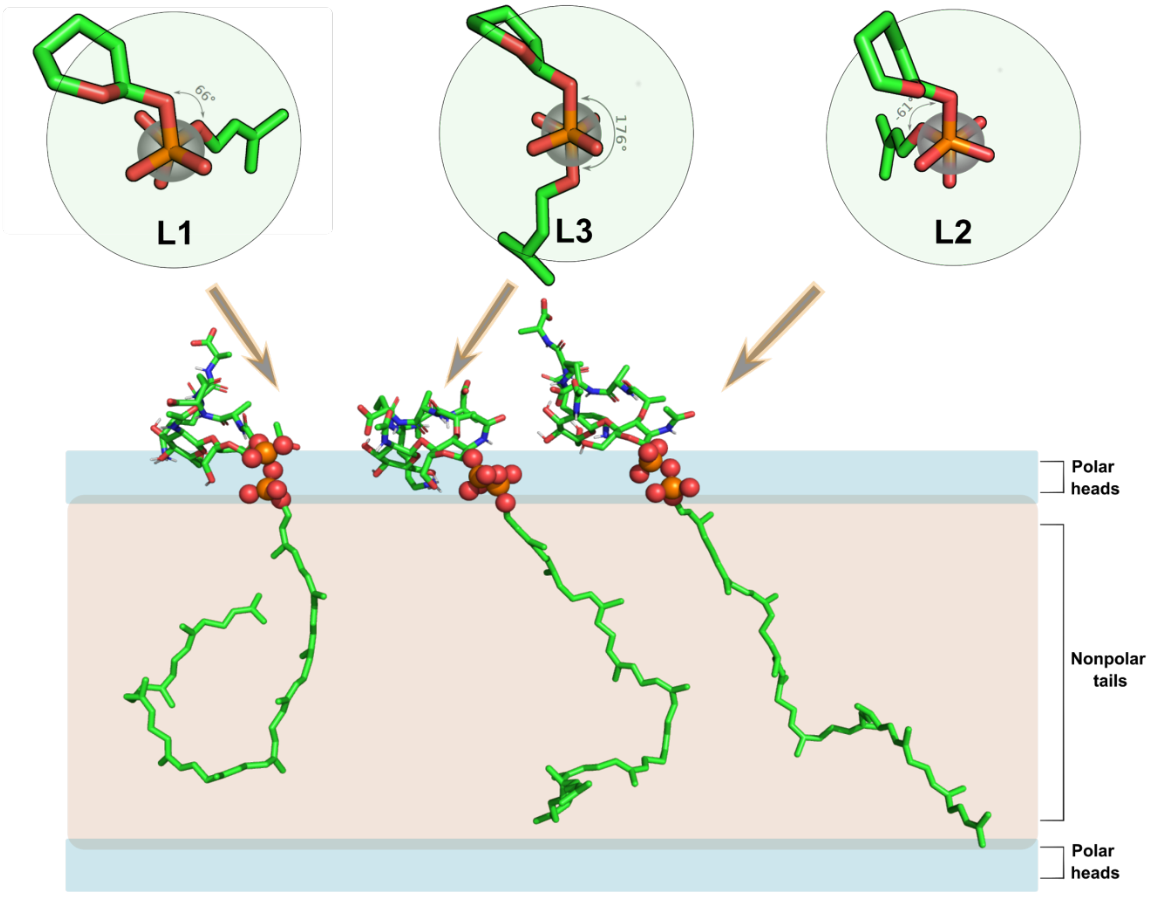


**Supplementary Figure S3. The set of lipid II's pyrophosphate conformations in the model membrane.** The snapshots of lipid II states with three different PPi dihedral angle (L1, L2 and L3) from MD simulations with zoomed in image depicting the projection of OPPO torsion. Note that the relative PPi orientation with respect to the bilayer normal is not determined by PPi states (L1-L3).

*L1, L2*: Hydrogen bond acceptors at unique distance are exposed on bilayer surface. The conformation is theoretically suitable for capturing by nisin.

*L3*: Solvent-exposed oxygen atoms are located at distances typical for other membrane lipids. In this conformation, the pyrophosphate pharmacophore is facing the lipids and therefore is unable to form the complex.

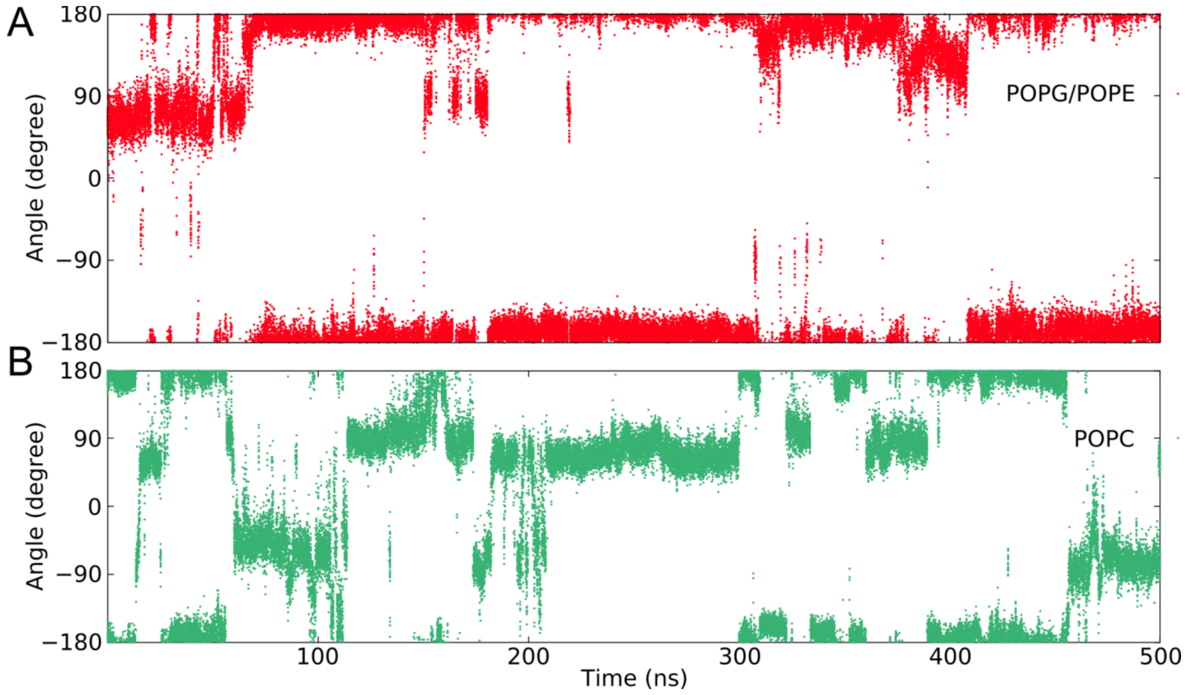


**Supplementary Figure S4. Evolution of lipid II's dihedral angle OPPO in different bilayers in the course of MD simulations.** Conformational transitions occur less frequently in POPG/POPE membrane **(A)** as compared to those in POPC membrane **(B)**.

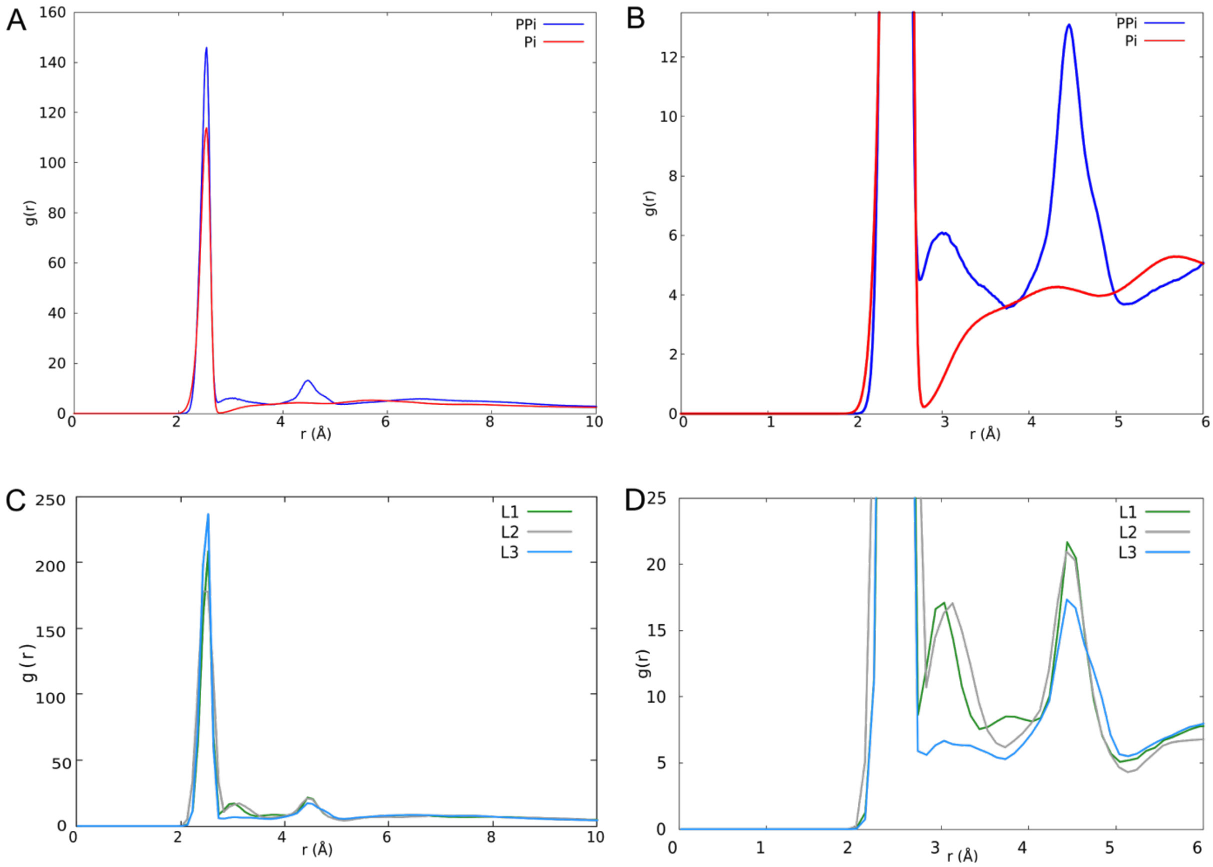


**Supplementary Figure S5.Oxygen–oxygen radial distribution function (gOO) for membrane-bound lipid II and phospholipids.(A)** gOO for all oxygen atoms in PPi (*blue*) and Pi (*red*) groups. **(B)** Zoomed-in fragment of gOO in PPi (*blue*) and Pi (*red*) groups reveals three maxima. **(C)** gOO for *solvent-accessible* oxygen atoms in different conformations of lipid II: L1 (*green*), L2 (*grey*), and L3 (*light blue*). **(D)** Zoomed-in fragment of gOO for *solvent-accessible* oxygen atoms in: L1 (*green*), L2 (*grey*), and L3 (*light blue*).

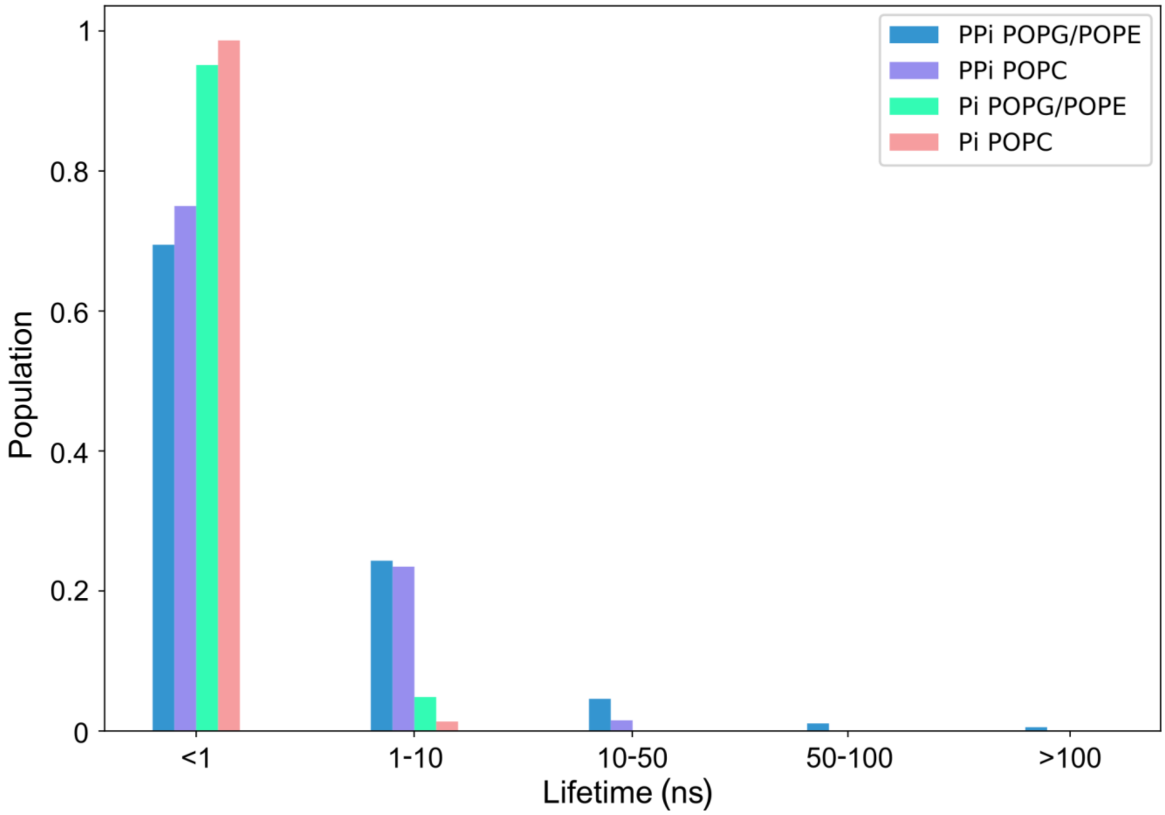


**Supplementary Figure S6. Distribution of the lifetime of pairs of solvent-accessible oxygen atoms at 3.0±0.1 Å distance in PPi and Pi groups: results of MD simulations**. The maximum lifetime of exposed at the surface oxygen pair in PPi is 140 ns and is achieved in POPG/POPE membrane, while in POPC bilayer it does not exceed 20 ns. At the same time, for Pi oxygen atoms lifetimes do not exceed 16 and 7 ns, in POPG/POPE and POPC, respectively. The timescale was calculated with tolerated gap of 0.5 ns. Data sets are normalized to one.

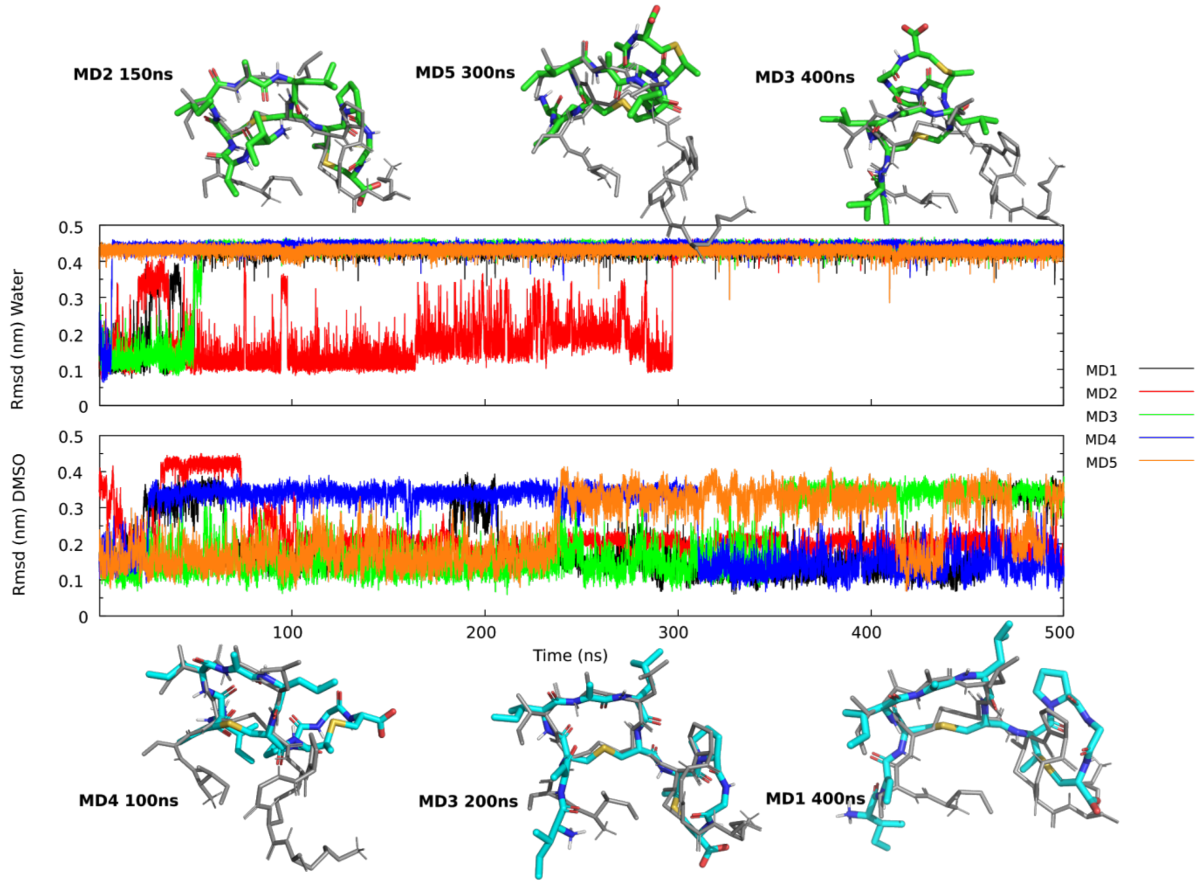


**Supplementary Figure S7. Backbone RMSD values of nisin$_{1–11}$ rings A and B were generated against the NMR structure of nisin / lipid II analogue complex in DMSO (PDB ID: 1WCO) during 500 ns of nisin$_{1–11}$/DMPPi complex MD simulation.** *Upper panel*: 5 MD trajectories in water solution. *Lower panel*: 5 MD trajectories in DMSO. Snapshots of the relevant complexes are shown at the top and bottom, respectively. Carbons of the nisin rings A and B are colored green and blue for water and DMSO simulations, respectively. Aligned by ring A backbone 1WCO nisin structure is shown with grey sticks.

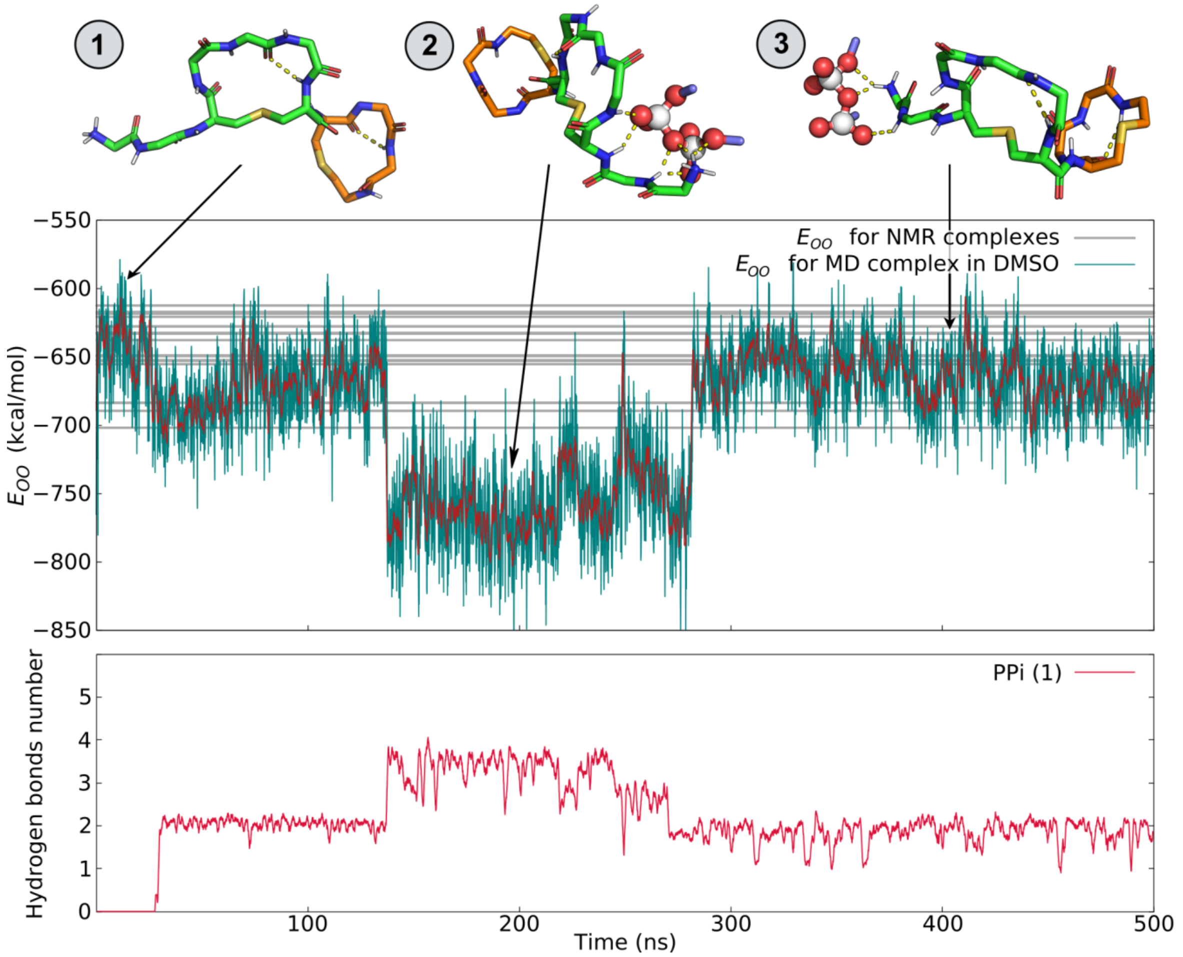


**Supplementary Figure S8. Energy of the pyrophosphate pharmacophore ($E_{OO}$) for nisin$_{1-11}$/ DMPPi complex spontaneously forming during MD simulation in DMSO.** *Upper panel:* $E_{OO}$ (instant and time-averaged (1 ns window) values are shown with *green* and *red lines*, respectively) as a function of MD time illustrates the process of the complex formation. *Grey lines* correspond to calculated $E_{OO}$ values for 20 NMR structures of nisin / lipid II analogue complex in DMSO. The relevant complex snapshots are shown on the top. Carbons of nisin rings A and B are colored *green* and *orange*, respectively. DMPPi is shown with *spheres* and *purple sticks*. *Lower panel:* The time-averaged (0.1 ns window) number of H-bonds between nisin$_{1-11}$ and DMPPi (*red*).

Note that $E_{OO}$ describes solely the ability of a given conformation of nisin$_{1-11}$ to form a complex with PPi, but not the DMPPi interaction energy. The low-energy complex is formed by four N-terminal residues (note the difference from stable complex #3 in water (Fig. 7), where the whole ring A takes part). The same complex structure was found in aqueous solution, however with higher $E_{OO}$ value (−680 kcal/mol; complex #1 in Fig. 7). The number of intermolecular H-bonds anticorrelates with $E_{OO}$ (R = −0.72).

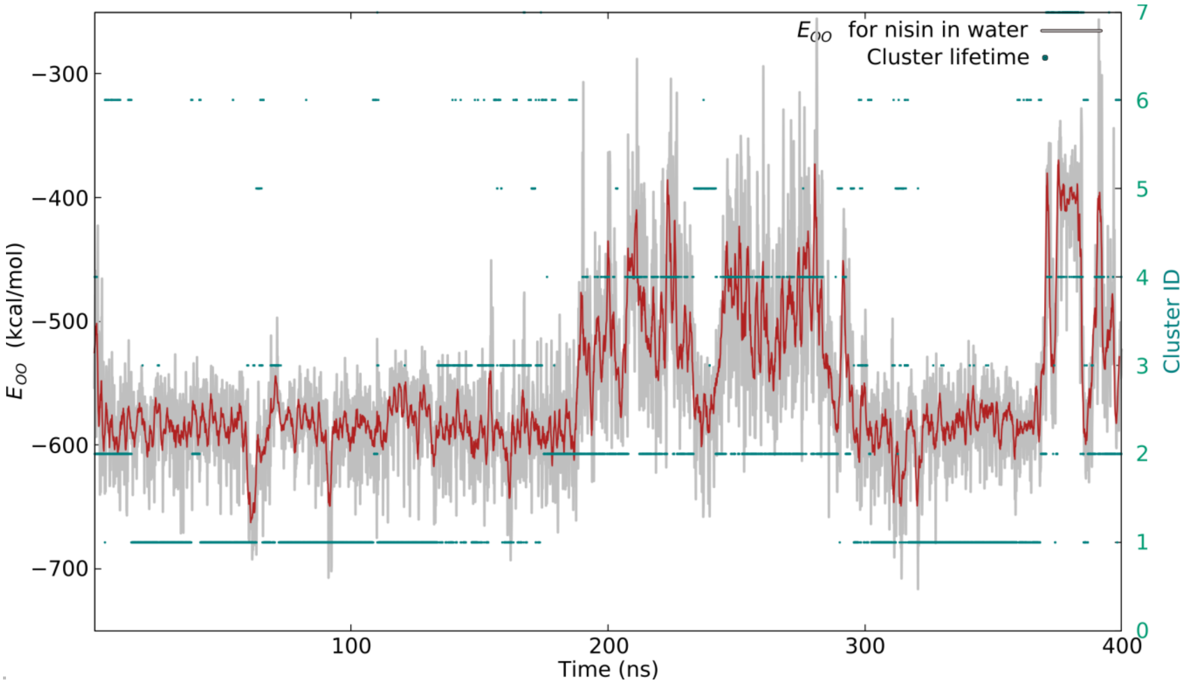


**Supplementary Figure S9. Energy of the pyrophosphate pharmacophore ($E_{OO}$) of isolated nisin$_{1–11}$ during MD simulation in water.** $E_{OO}$ values with step of 100 ps and averaged by 1 ns are shown with *grey* and *red lines*, respectively, as a function of MD time (*left axis*). *Green dots* correspond to nisin$_{1–11}$ conformational cluster: N1–6 (*right axis*; see Fig. 5 for clusters description).

$E_{OO}$ revealed two major states: preformed target trap (united nisin$_{1–11}$ states N1, N3, N5, N6) and "inactive" form (N2 and N4). The low energy values (below −683 kcal/mol, corresponding to "stable" MD complex in water (table 1)) are inherent to the closed-ring conformation and put together 0.43% of the calculated trajectories.

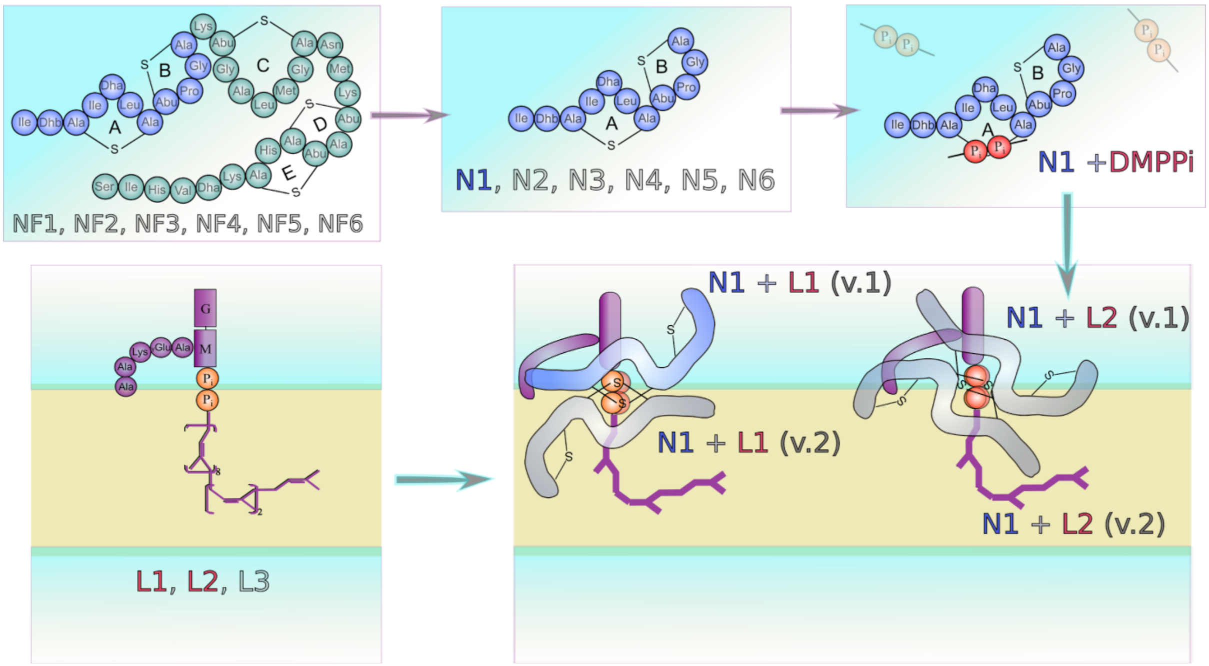


**Supplementary Figure S10. Schematic representation of different conformations of studied molecules revealed in the MD simulations.** States that are not capable of binding are shown in *grey*; competent conformations are highlighted with *blue* (nisin$_{1-11}$) and *red* (lipid II). Based on the found competent conformations of both molecules (N1, L1 and L2), four possible models of their complex in the membrane were constructed: by fitting L1 or L2 states of lipid II to DMPPi (in N1-DMPPi complex), each in two variants due to symmetry of DMPPi. Only one N1-L1 was not sterically hindered (N1+L1 (v.1), *blue*). The rest combinations are shown *grey* due to overlap with explicit membrane lipids.

**Supplementary Table S1. Percentage (%) of the first cluster occurrences along given MD replica (N1 and NF1 for nisin$_{1\text{-}11}$ and nisin, respectively).**

| | nisin$_{1–11}$ | nisin |
|---|---|---|
| MD 1 | 47.8 | 1.3 |
| MD 2 | 48.6 | 10.9 |
| MD 3 | 76.2 | 75.2 |
| MD 4 | 1.1 | 87.6 |
| MD 5 | 2.62 | 74.4 |

Note that nisin cluster analysis was performed using merged data from all MD trajectories (for full-length peptide and nisin$_{1–11}$ separately). However, the analysis parameters were adjusted to generate an ensemble of nisin structures with each of 3 top clusters containing frames from at least 3 of 5 replicas. Table S1 demonstrates reproducibility of cluster analysis.

**Supplementary Video S1.** *Structure of stable nisin$_{1–11}$ / DMPPi complex in water solution.* First seven seconds show *nisin$_{1–11}$*structure with overlaid spatial density of "energy of the pyrophosphate pharmacophore" ($E_{OO}$), illustrating the optimal location of the probe oxygen atoms pair. The last ten seconds additionally show DMPPi position. Rings A and B of nisin are represented as *sticks* and colored *green* and *orange*, respectively. The volume surface indicates predicted density-colored positions of the O–O pharmacophore (where *blue* and *red* colors correspond to the least and most occupied positions, respectively), interacting most favorably with a given nisin$_{1–11}$ conformation (according to $E_{OO}$ criterion). DMPPi is shown with *purple sticks*. The oxygen atoms of PPi moiety observed in MD simulation inscribe well into this predicted density.

The movie is availible in the Zenedo archive (https://doi.org/10.5281/zenodo.3572677).
